# Murasaki: A Fast, Parallelizable Algorithm to Find Anchors from Multiple Genomes

**DOI:** 10.1371/journal.pone.0012651

**Published:** 2010-09-24

**Authors:** Kris Popendorf, Hachiya Tsuyoshi, Yasunori Osana, Yasubumi Sakakibara

**Affiliations:** 1 Department of Biosciences and Informatics, Keio University, Yokohama, Japan; 2 Department of Computer and Informatics Science, Seikei University, Musashino-shi, Tokyo, Japan; Institute of Infectious Disease and Molecular Medicine, South Africa

## Abstract

**Background:**

With the number of available genome sequences increasing rapidly, the magnitude of sequence data required for multiple-genome analyses is a challenging problem. When large-scale rearrangements break the collinearity of gene orders among genomes, genome comparison algorithms must first identify sets of short well-conserved sequences present in each genome, termed anchors. Previously, anchor identification among multiple genomes has been achieved using pairwise alignment tools like BLASTZ through progressive alignment tools like TBA, but the computational requirements for sequence comparisons of multiple genomes quickly becomes a limiting factor as the number and scale of genomes grows.

**Methodology/Principal Findings:**

Our algorithm, named Murasaki, makes it possible to identify anchors within *multiple* large sequences on the scale of several hundred megabases in few minutes using a single CPU. Two advanced features of Murasaki are (1) adaptive hash function generation, which enables efficient use of arbitrary mismatch patterns (spaced seeds) and therefore the comparison of multiple mammalian genomes in a practical amount of computation time, and (2) parallelizable execution that decreases the required wall-clock and CPU times. Murasaki can perform a sensitive anchoring of eight mammalian genomes (human, chimp, rhesus, orangutan, mouse, rat, dog, and cow) in 21 hours CPU time (42 minutes wall time). This is the first single-pass in-core anchoring of multiple mammalian genomes. We evaluated Murasaki by comparing it with the genome alignment programs BLASTZ and TBA. We show that Murasaki can anchor multiple genomes in near linear time, compared to the quadratic time requirements of BLASTZ and TBA, while improving overall accuracy.

**Conclusions/Significance:**

Murasaki provides an open source platform to take advantage of long patterns, cluster computing, and novel hash algorithms to produce accurate anchors across multiple genomes with computational efficiency significantly greater than existing methods. Murasaki is available under GPL at http://murasaki.sourceforge.net.

## Introduction

“Homology search” plays a fundamental role in a variety of sequence analysis studies. The goal of a homology search is usually some form of the *Longest Common Subsequence* (LCS) problem. In the most general form, with an unbounded number of sequences, LCS is an NP-Complete problem, therefore any attempts to solve the problem quickly and at scale are forced to recognize only a limited subset of the problem. Limiting the number of sequences under comparison to some fixed number 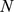 allows the now ubiquitous Smith-Waterman polynomial-time dynamic programming solution [1] to be used. Like most NP-Complete problems, what is easy for a few small objects becomes impractical for larger more numerous objects. Indeed the time and space requirements of Smith-Waterman are considered prohibitive for large (or more critically numerous) sequences, leading to the evolution of modern homology search algorithms that employ some heuristic to provide an approximation of the exact LCS solution. Newer algorithms like FASTA [2] and later BLAST [3] and its derivatives (PatternHunter, BLASTZ, Mauve, etc.) rely on subsequences of unusually high conservation to “anchor” a search to a smaller area where a more detailed homology search can be conducted in reasonable time, from which the term *anchor* is derived. The increasing availability of sequences and the now common need to align multiple whole genomes has repeatedly pushed each of these homology search algorithms to the point where they are no longer viable, demanding the development of software that takes advantage of new technologies and novel algorithms with refined heuristics. Our software, Murasaki, is yet another entry in this tradition. We also follow the UNIX tradition of making a tool to do one job and do it well. Thus we confine the scope of Murasaki to that of *anchor search* on *multiple genomes* only, and leave the question of what to do with anchors to other tools further down the toolchain. Our goal was to create an efficient flexible way to search for anchors that meet arbitrary constraints across multiple genomes (as opposed to simple pairwise comparisons) while taking advantage of the increasingly multi-core and distributed computational environments available to researchers.

### Anchoring

The term “anchor” generally refers to well-conserved short regions among two or more genomes, and is biologically defined as a short gene-coding region or an exon in a long gene or non-coding region (including functional RNAs) where no rearrangement occurs. Computationally, anchors are generally determined by identifying occurrences of matching *k-mers* and extending or combining them as high scoring pairs.

The task of finding anchors is considerably different from producing alignments. Finding anchors is only the first step of BLAST [3], in that BLAST produces lots of anchoring pairs and tries to extend them. Mauve [4], for example, relies on anchors only for finding the endpoints of alignable collinear regions. Therefore, anchoring alone is not expected to be as sensitive as exhaustive gapped-alignment, but anchoring multiple genomes can rapidly yield information that can be used to reduce the computation time of multiple genome alignments [5], to infer genome rearrangements through synteny identification [6], to find conserved non-coding RNA regions which are usually much shorter than protein-coding regions, and to execute genome-wide evolution analysis such as the identification of ultraconserved regions [7].

### Previous Work

Modern homology search programs generally rely on some efficiently searchable data structure to index the locations of short subsequences (we will call these subsequences “seeds”). There have been many approaches to doing this. Mauve [4] uses a sorted list that is simple and space efficient, and because Mauve prunes all but the unique seeds, usually fast. MUMmer [8] and later ramaco [9] use suffix trees to find short exact matches. The latter implements a pairwise comparison based approach to finding matches across multiple sequences while relaxing the “unique” constraint of *multiMUMs*, however offers little opportunity for parallelization and is limited by the space requirements of its tree structures. The speed gains from FASTA/BLAST and the vast majority of popular modern derivatives such as BLASTZ [10] come from storing the seed index in a hash table where look-up of a given seed is constant time. In practice this hash table is generally a block of contiguous memory in the computer, such that we might think of it as a table of 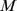 entries, 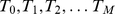. Key-value pairs 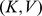 might then be recorded in the table by storing 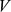 in the entry 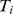 specified by a *hashing function* 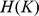 (i.e., where the *hash* 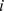 of 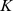 is defined by 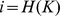). For homology-finding, the key 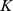 would generally be a “seed” (e.g., ATGC), and the value 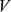 would be the location in the input sequence(s) at which it occurs. Because ATGC might occur any number of times, hash table entries are often some list-like data-structure that allows a different 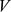 to be stored for each incidence of the same seed 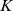. The performance of a hash table then depends on the ability to find the entries that match a given key quickly. In other words if 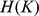 is slow, or storing to and retrieving from 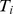 is slow, performance deteriorates. Ideally 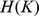 produces a different hash 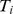 for every different value of 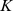, but when two keys 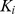 and 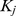 such that 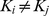 produce the same hash (i.e., 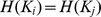) separating their values 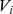 and 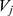 in the hash table requires additional work. These events are called “collisions.” Thus to minimize the time spent resolving collisions, the selection of a hash function 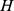 that avoids collisions is at least as important as how to resolve them. In cases where the maximum number of keys is small, as in PatternHunter and BLASTZ where keys are at most 12 or 14 bases (limiting the number of possible keys to 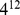 or 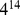 respectively), the size of the hash table 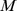 can be chosen to accommodate all possible keys, and the hash function 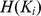 can simply be the position of 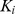 in an enumerated list of all possible values of 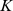 (if we think of a string of nucleotides as a base 4 number, thus 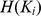 becomes the trivial identity function 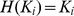). This is the standard method used by most existing hash-based homology search algorithms, and is acceptable for a small number of keys. However the size of the hash table required to guarantee no collisions increases exponentially with the length of keys (e.g., when using longer *k-mers*). Given 14 bases alone requires 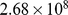 entries, which at even a modest 32 bits per entry is 1GB of memory, 15 bases requires then 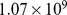 entries and 4GB, 19 bases requires 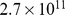 entries and 1TB and so on, it's obvious that if one wants to use longer keys a different solution is required. BLAST and BLASTZ limit this exponential expansion by using only the first 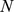 bases as a key when the key length exceeds a predefined threshold.

Ma *et al.* introduced the notion of *spaced seed patterns* to homology-search in PatternHunter [11]. Spaced seed patterns are typically represented as a string of 1s and 0s, where 1s represent bases that contribute to a “seed” and 0s do not. For example given the pattern 1011 and the sequence “ATGC”, we could generate two seeds, “A.GC” and “G.AT” (the reverse complement) where the “.” (period) characters are disregarded or can be thought of as matching anything, as in regular expressions. The *weight* of a pattern refers to the number of 1s in the pattern. Ma *et al.* showed that a spaced seed pattern is more sensitive to weak similarities than a non-spaced seed pattern of the same weight, leading to a small revolution in homology-search as programs were modified to incorporate spaced seeds. Calculating so called “optimal seed patterns” becomes a challenge for long seeds [12]. In general, however, shorter and lighter patterns are expected to be more sensitive while longer and heavier patterns are expected to increase specificity. The use of spaced seeds complicates the generation of hash functions, often limiting the choice of patterns (for example, BLASTZ offers users the choice of 2 spaced seed patterns).

When the MegaBLAST and BLASTZ approach of hashing using only the first 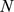 bases is applied to spaced seed patterns, we refer to this as the *First-N* approach. To examine the situations in which this *First-N* approach is suboptimal, considering genome hashing from a Shannon entropy perspective is helpful. Because a weight 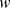 seed 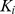 has at most 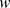 random symbols from an alphabet of {A,C,G,T}, 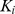 has at most 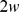 bits of information. The naive *First-N* approach to getting an 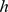 bit hash out of a 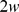 bit seed is simply to start reading bits from one end and stop once 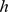 bits are collected, as described above for BLAST and BLASTZ. If each base were statistically independent, any sampling of 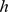 bits from the key 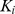 would be equally effective. In reality, however, each base of a *k-mer* is far from statistically independent. In fact, the average conditional entropy of a base genome given the previous bases is estimated to be closer to 1 bit per base [13]–[14]. Therefore, the naive approach is expected to provide poor utilization of the available hash key space. We confirm this in the Results section. At the other end of the complexity spectrum, we can expect near uniform utilization of the hash key space by passing all 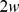 bits to a cryptographically secure pseudorandom hash function like SHA-1 [15] or MD5 [16]. SHA-1 and MD5 are often used as hash algorithms where the characteristics of the key domain are unknown and uniform utilization of the hash range is critical (such as in file systems [17] to prevent data loss). MD5 has recently been shown to be vulnerable to a variety of cryptanalysis attacks designed to generate colliding keys for a given key rendering it unsuitable for security purposes; however MD5 is faster than SHA-1 and the cryptanalytic attacks are irrelevant to our purposes here. These cryptographic hash functions are, however, computationally expensive and produce 256 bit hashes from which we can use only a small fraction. In Murasaki we introduce a novel hash function generation algorithm to automatically generate hash functions from arbitrary spaced seed patterns that approximate maximal hash key space utilization in a computationally inexpensive manner, which we term the “adaptive hash algorithm.” The details of this algorithm are explained in the Methods section.

### Motivation

Identification of anchors (or seeds for alignment) for whole-genome comparison plays a fundamental role in comparative genomic analyses because it is required to compute genome-scale multiple alignments [18]–[20], and to infer among multiple genomes orthologous genomic segments descendended from the common ancestor without any rearrangement [21]. A common approach for the identification of anchors among multiple sequences, used by TBA [22], first detects anchors between every pair of sequences, and then progressively integrates pairwise anchors to form anchors across multiple sequences. For a given number of sequences (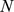), this approach requires 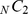 computations of pairwise anchors. Naturally this *progressive approach* requires quadratic (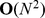) time with respect to the number of sequences. Linear time variations on this approach exist when alignments to a single reference sequence are appropriate (eg. the UCSC human conservation track [23]), however these have their own limitations which we describe in *Discussion*. Current progress in sequencing technologies accelerates the accumulation of completely sequenced genomes: 1,139 Prokaryotic and 129 Eukaryotic genomes are now available as of this writing (6th May, 2010) according to the GOLD database [24]. The rapidly increasing number of available genomes poses a scalability challenge for bioinformatics tools where computational cost is bound to the number of sequences. Progressive alignment is further complicated by the potential to introduce errors or bias based on the phylogenetic trees selected for progressive alignment and accumulate pairwise errors at each stage of the alignment [25]. To address these issues, we propose an alternative to the progressive approach allowing the identification of multi-sequence anchors simultaneously wherein all sequences are hashed simultaneously and well-conserved anchors are computed in a single pass. This allows us to compute anchors across multiple genomes with an approximately linear cost without any pairwise comparisons or tree inference.

### Parallelization

With processor densities now pushing the constraints of physics for speed [26], chip manufacturers have abandoned increasing clock speeds in favor of adding multiple cores and increased parallelism. To deal with the exponentially increasing amount of sequence data available from new sequencing technologies [27], new algorithms need to be designed to take advantage of multicore and cluster computing environments in order to keep up. Furthermore, conventional computer architectures impose a strict limit on the amount of maximum amount of RAM usable in a single machine. This has pushed developers working on whole genome data, as with ABySS [28], to use cluster computing to avoid memory barriers even if there is little gain in computational speed (or even a decline).

When using progressive alignment tools such as BLASTZ and TBA, the 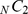 comparisons for each pair of sequences are independent and therefore trivially parallelizable. Any finer grained parallelization (necessary to use more than 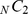 processors), requires breaking the 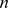 sequences into smaller fragments that can be aligned independently (the technique used for Human and Mouse genomes in [10]). This fragmentation is in fact necessary with software like BLASTZ for mammalian scale genomes where the genome as a whole is too large to be processed by the alignment software in a single pass. Breaking each sequence into 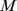 fragments incurs an additional cost for each pair 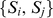 of the 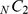 comparisons. With fragmentation each comparison is shorter, however each base is considered at least 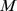 times more than it was without fragmentation. This is because all 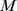 fragments of sequence 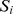 must be compared with all 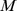 fragments of sequence 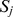 and each fragment gets re-indexed and anchored each time. In Murasaki we eliminate both the 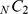 and fragmentation costs by introducing a novel fine grained yet highly efficient approach to parallel anchoring using an unlimited number of processors independent of the number of sequences or fragments under comparison.

## Methods

### Algorithm Outline

At its most primitive definition, Murasaki takes as input a set of DNA sequences, a *spaced seed pattern*, and provides as output a series of *anchors*.

**Figure 1:**
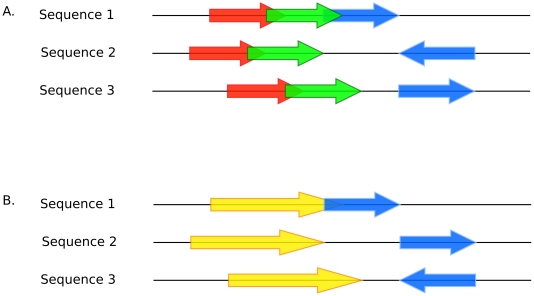
Anchor coalescing. Here we illustrate an example of how anchor coalescing is processed. In **A** we show 3 anchors spanning 3 sequences represented by 3 sets of arrows: red, green, and blue. The green anchor overlaps the red anchor in all sequences, and maintains colinearity, therefore they can be coalesced. However the overlap of green and blue occurs only in Sequence 1, therefore they cannot be coalesced. **B** shows the results of the coalescing of green and red with the resulting anchor shown in yellow.

*Anchors* are defined in Murasaki as a set of intervals across some subset of the input sequences. Each anchor contains at least one set of matching *seeds*. Here a *seed* refers to an input substring when masked by the *spaced seed pattern*. When an anchor is initially constructed based on a set of matching seeds, both ends are extended by an ungapped alignment until the minimum pairwise score falls below the X-dropoff parameter as in BLAST and BLASTZ [10]. Overlapping collinear anchors are coalesced to form larger anchors, as in Figure 1.

Because our goal is to find and extend matching seeds, the role of the hash table is to accelerate the identification of matching seed sets. FASTA, BLAST, and BLASTZ all rely on hash table-like indices to find matching seeds in constant time. Mauve uses a “sorted k-mer list” where *k-mers* (or in later versions pattern masked *k-mers*) are stored in a list and sorted. Suffix trees and other tree-based approaches use some tree-like structure to accomplish the sorted index task. As described in Previous Work, the strict one-to-one hash table-like approaches in the FASTA derivatives limit the size of seeds to 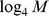 where 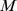 is the size of the hash table. Murasaki uses a hybrid approach mixing hash tables with a fast comparison based collision resolution mechanism to reduce the number of comparisons needed to find matching seed sets. Hashes are generated from seeds such that if two seeds 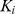 and 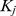 match, they necessarily produce the same hash (i.e., 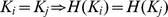), therefore all matching seeds will reside in the same location within the hash table. Collisions are resolved by either using chaining and a sort or by open addressing.

The algorithm is as follows:

1. Load the input sequences as 2-bit codes.
2. Determine hash parameters and hash function 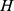.
3. For each location 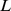 across all input sequences (on both forward and antisense strands):
  - Compute a hash 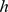 for the seed 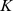 at location 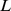 based on the input sequence and hash function 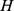

  - Store the pair 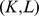 into location 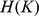 in the hash table.
  At this point all matching locations (“seeds”) share the same hash key and therefore share the same locations in the hash table.
4. Extract all matching sets of seeds from each entry of the hash table (i.e., “invert” the hash table).
5. For each set of matching seeds:
  - Make a new anchor 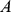 for each subset of seeds such that there is exactly one seed from each sequence.
  - Extend each new anchor 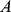 by ungapped alignment.
  - Coalesce each new anchor with pre-existing existing anchors.

**Figure 2:**
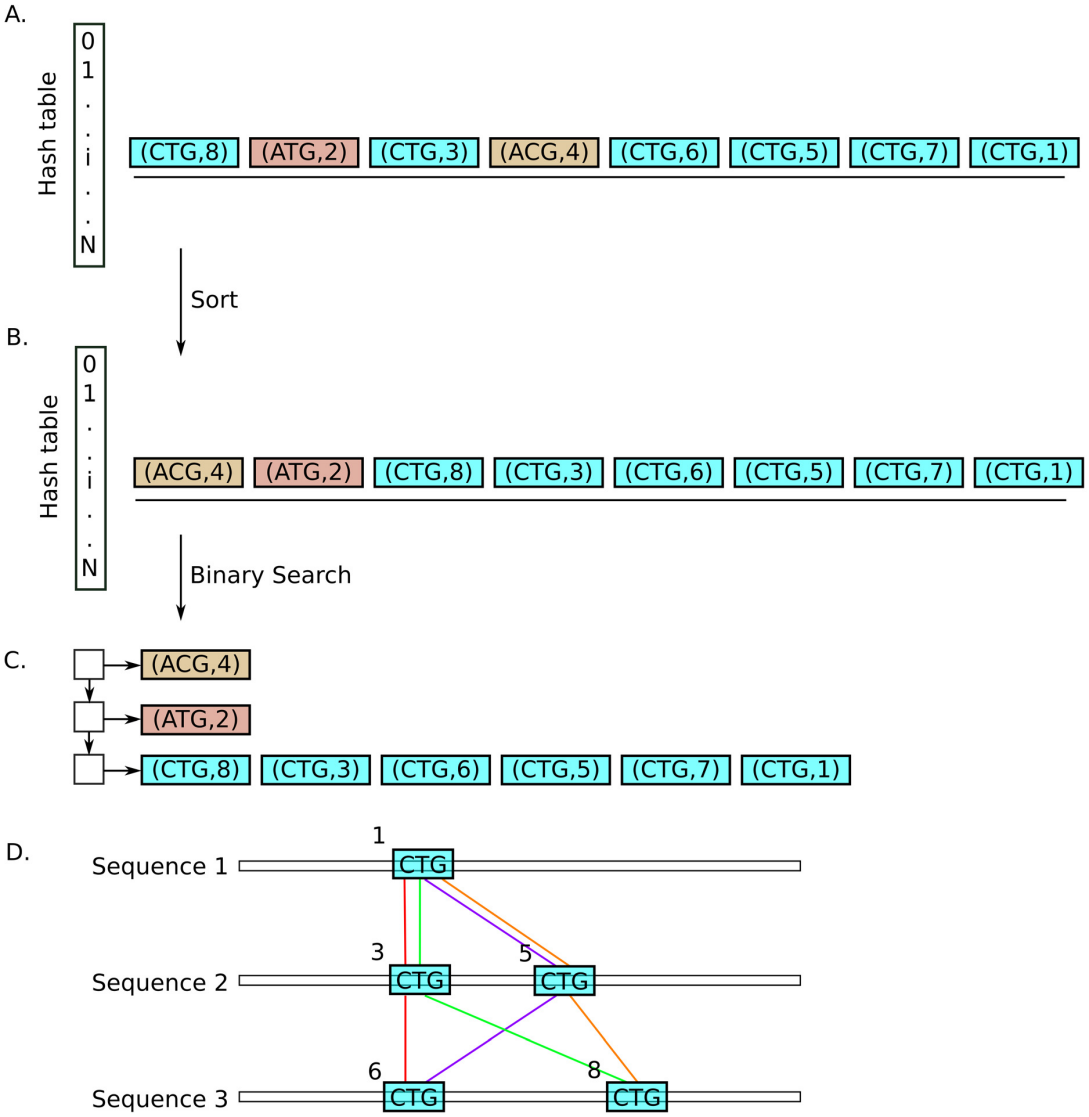
Hash table inversion and anchoring. Here we show a simplified example of how matching seed sets are extracted from the hash table and converted into anchors. **A** shows one row (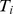 out of 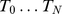) of the hash table. Several 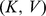 pairs have been inserted into the hash table. 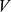 indicates a position in the input sequences at which 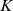 occurs. Because 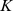 is necessarily implied by 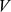 Murasaki only stores 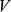, however here both 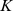 and 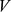 are shown for clarity. Different values of 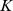 have also been colored differently to note their difference. First, this row is sorted with the result shown in **B**. The extents of each matching seed set can then be found in 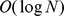 time by binary search. These matching seed sets are extracted into a series of lists, as shown in **C**, which are then used to construct anchors, as shown in **D**.

The hash table inversion and anchor generation steps are illustrated in Figure 2. Murasaki optionally supports partial matches, also known as “islands” where some number of sequences may be missing. In this case 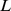 for sequences up to the specified number of missing sequences are considered anchored at a special 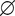 location.

It is worth noting at this point that the size of the hash table is a critical factor. Our hash table size is defined to be exactly 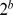 where 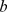 is the “hashbits” parameter, describing the number of bits expressed in hash values. The events where 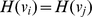 and 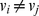 are termed “hash collisions.” While careful selection of a hash function can reduce the number of hash collisions, the pigeon hole principle guarantees that some collisions must occur if the number of distinct seeds is greater than the size of the hash table. Even given a perfectly balanced hash function, where a seed selected at random has an equal probability of mapping to any key, the expected number of collisions per key is 
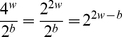
, where 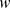 is the weight of the pattern. Therefore, increasing the value of 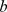 by one is expected to reduce the number of collisions by half, dramatically reducing the time required to invert the hash table and extract matching seeds. This trade-off of memory for speed is common in hash tables and in data structures overall, but Murasaki is the only existing hash-based anchoring algorithm to separate the selection of spaced seed patterns from the data structure used to index the input sequences. This gives the user separate tunable parameters that allow control of sensitivity/specificity independent of the memory footprint on the system.

### Parallelization

Murasaki's approach lends itself to parallelization at several points. First, the order in which individual seeds are hashed is irrelevant, and therefore we can devote as many CPUs to hashing as desired. The storage of locations into the hash table may require traversing and updating some form of list or tree structure, and which takes time comparable to that of computing a hash. Only one CPU can modify a list or tree at a time; however, if the lists are independent, CPUs can work on independent lists or without risk of interfering with each other. “Inverting” entries in the hash table can also occur in any order, and the more CPUs we apply to this task the faster it will finish. Therefore we divide all available computational nodes into one of two disjoint sets: “hasher nodes” and “storage nodes.” These nodes function in a “producer/consumer” model where one set performs one half of an operation and passes the result to a node in the opposite set. Fundamentally the parallel algorithm works as follows:

1. All nodes load input sequences as 2-bit codes.
2. Hash parameters and a hash function 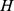 are generated.
3. Nodes are assigned a job as either “hasher” or “storage.”
4. The input sequence is divided into contiguous segments, one for each hasher.
5. Storage nodes are assigned a contiguous interval of the hash table to manage.
6. Each hasher node
  - computes a hash 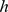 for the seed 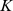 at location 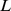 based on the hash function 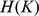.
  - sends this 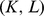 pair to the storage node responsible for 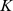.
7. Meanwhile, each storage node
  - receives a 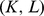 pair from a hasher node.
  - stores 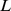 into location 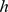 within the hash table.
8. Hasher and storage nodes now switch roles, the storage nodes becoming producers, and the hasher nodes becoming consumers.
9. Each storage node
  - inverts one row of the hash table at a time.
  - sends the each resulting set of matching seeds to an arbitrary hasher node.
10. Meanwhile each hasher node, now maintaining an independent set of anchors
  - receives a set of matching seeds from a storage node.
  - makes a new anchor 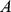 for each subset of seeds such that there is exactly one seed from each sequence.
  - extends each new anchor 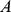 by ungapped alignment.
  - coalesces each new anchor with pre-existing anchors.
11. Once all hasher nodes have finished receiving and building anchors, hasher nodes have to merge these anchors between them. This is the “distributed merge” step. Initially all hasher nodes contain unmerged anchors and are considered “active.”
12. Active hashers are broken into “sender/receiver” pairs, such that hasher 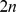 receives anchors from hasher 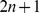.
13. Anchors are merged by the receiver into the pre-existing anchor set, just as new anchors were in the sequential algorithm.
14. Hashers that have finished sending are deactivated, and the remaining hashers repeat the process from step 12 until all anchors reside on a single hasher.

**Figure 3:**
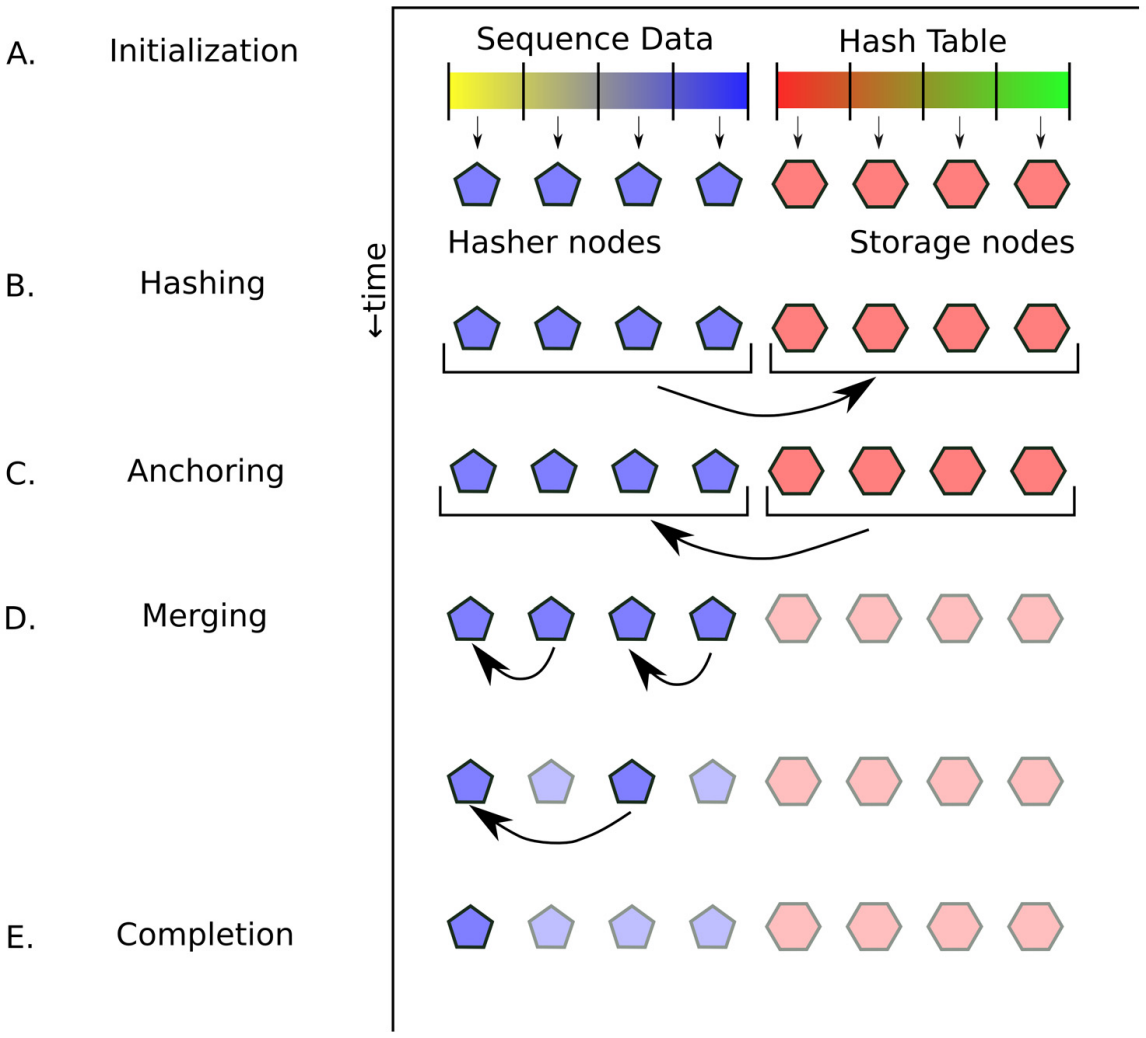
Parallel algorithm overview. Here we show a simplified 8 node example of the parallel Murasaki algorithm. The time axis shows the progression of steps and is not drawn to scale. At **A** Each hasher node is assigned an equal part of the sequence data (depicted as yellow-blue line), and each storage node is assigned a part of the hash table (depicted as a red-blue line). At **B** nodes have been divided into “hasher nodes” (shown as blue pentagons) and “storage nodes” (shown as red hexagons). Here hasher nodes act as producers, hashing the input sequence and passing 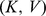 pairs to the storage nodes which store them in the hash table. At **C**, the producer/consumer roles are switched such that storage nodes extract matching seed sets from the hash table and send them to hash nodes. Once all matching seed sets have been extracted, the storage nodes are finished and can be terminated (indicated by the lighter coloring in **D**). At this point each hasher node has an independent anchor tree. Hasher nodes are divided into pairs, with one node sending all of its anchors to the other. These anchors are merged using the normal coalescing algorithm. Once a hasher has finished sending, it can be terminated. Because the number of hasher nodes is halved at each iteration, this merge step finishes in 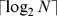 iterations, where 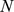 is the number of hasher nodes. At **E** only one hasher node remains which handles any additional scoring and filtering of anchors, and outputs the final result.

The final “distributed merge” step (11 above) is unique to the parallel algorithm, and is the only place where additional overhead for parallelization is introduced. The memory overhead is minimal, and because the number of active hashers is halved at each iteration, the distributed merge step requires only 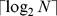 (where 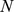 is the number of participating hasher nodes) iterations to complete. The parallel algorithm is summarized in Figure 3.

Most modern workstations and servers used in cluster environments generally have a limited amount of RAM available. Therefore, Murasaki's parallelization scheme presents a useful advantage in that it that allows the biggest memory requirement, the hash table, to be broken up across an arbitrary number of machines. This enables the use of proportionately larger hash tables and thereby enables fast indexing of larger sequences such as multiple whole mammalian genomes. Murasaki automatically exploits this increased available memory by incrementing the hashbits parameter (doubling the size of the hash table) each time the number of machines available in a cluster doubles.

### Hash function generation

As described above, the choice of hash function determines the efficiency with which the hash table can be utilized. Therefore, it is important that the hash function be chosen with care. The ultimate goal of the hash function is to provide a means of reducing the number of operations necessary to identify all seeds matching a given seed 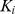. Therefore, we would like each key of the hash table to be shared by as few seeds as possible. The “ideal” hash function provides minimal collisions while requiring only minimal computation to calculate 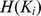.

**Figure 4:**
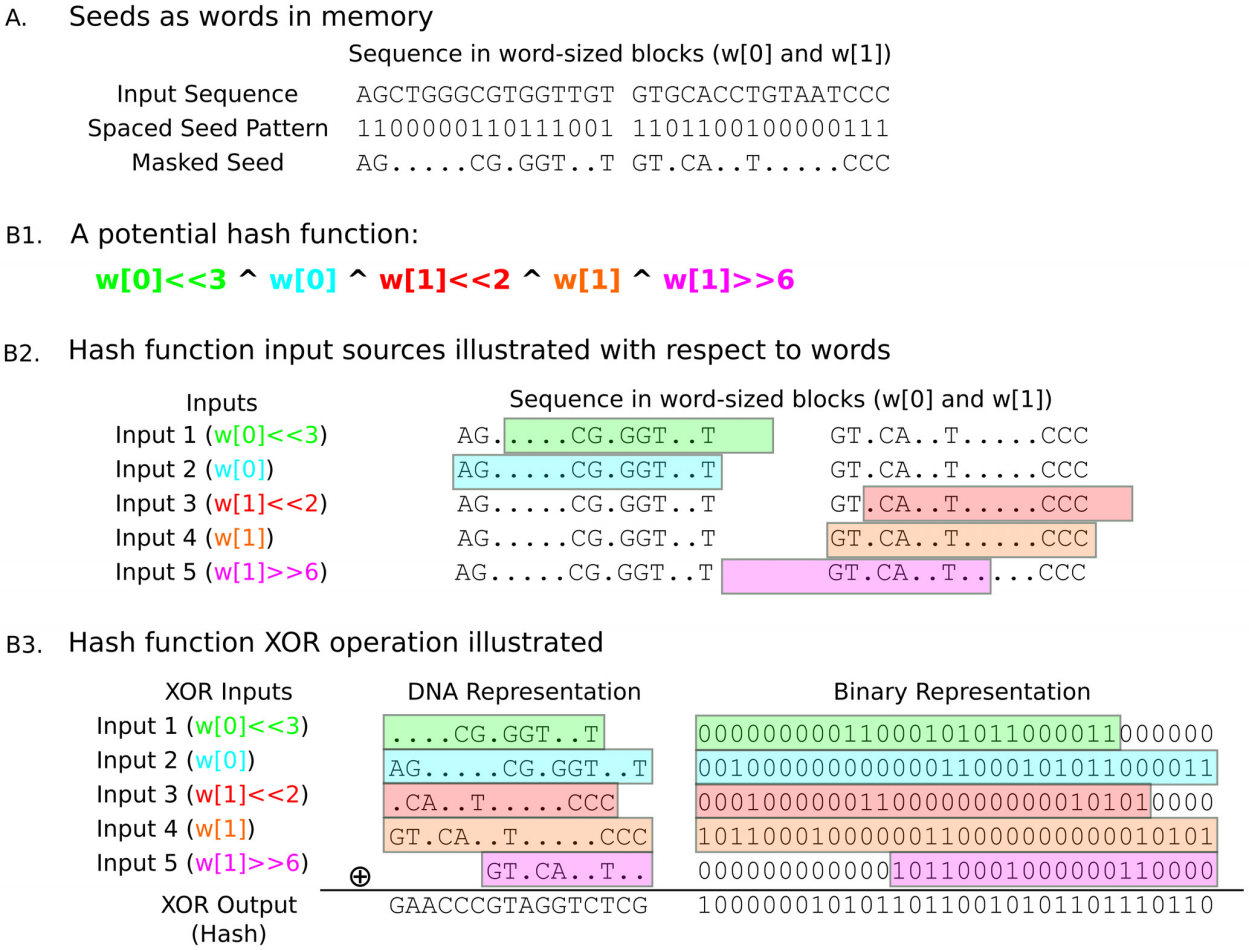
Hash calculation. Here we show an example hash function and how it is calculated. **A** illustrates how a seed might be stored in memory on a 32-bit machine. We show 32 bases of sequence stored in two 32-bit (16 base) words. The spaced seed pattern we're using as an example here is 32 bases long, with a weight of 16. The last line of **A** shows the input sequence after being masked by the spaced seed pattern, where the masked bases have been replaced with. (periods). **B1** shows an example hash function, expressed in C terms as a series of words (w [0] or w [1]), in most cases bit shifted left (≪) or right (≫), and conjunctively XOR'd together (the ∧ operator). **B2** shows the section of sequence being selected by each bit shifted XOR term. **B3** shows the actual XOR calculation that takes place with each bit shifted term expressed both as DNA bases and in binary. The highlighted regions show positions in the hash affected by the input sequence, with the color indicating the XOR term from which they originated. The final resulting hash is shown on the bottom line.

To describe our adaptive hash algorithm, first recall that input sequences in Murasaki are stored with two bits per base. Thus a “word” (the most primitive computational unit on which a CPU can operate) in a modern 64-bit CPU contains 32 bases, and a 32-bit word would contain 16 bases; however the algorithm itself works with any arbitrary word size 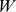. The *spaced seed pattern* is also expressed in the same two bits per base format, and therefore consists of several words 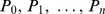, where 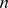 is number of words required to express the pattern (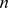 is therefore 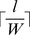 where 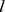 is the pattern length). An example seed and resulting words are shown in Figure 4A. Hashing by any of the above algorithms requires first that the bases ignored by the spaced seed pattern (the 0s) are masked (or eliminated). This can be accomplished for any given location 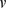 in the input sequence by simple bitwise AND operation. Because this operation will be repeated for each position in the genome, a pattern-sized buffer (which we call a *window*) of 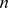 words (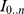) is prepared to facilitate this calculation. 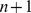 words from 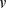 are copied into the window and bit shifted to align 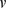 to the initial word boundary. The spaced seed masked word 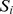 can be computed as the simple bitwise AND of 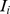 and 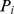. When hashing the whole input sequence, after hashing one window, the next window can be calculated by again simply bit shifting each word 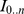 and recalculating the bitwise AND for 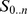.

This provides a framework for running arbitrary hash algorithms on spaced seeds. However no single one of these words alone is likely to make a good hash, as the masked bases in them provide zero entropy, and because the other bases aren't expected to be conditionally independent. To maximize the entropy of the hash, it is useful to combine words from across the breadth of the pattern. Therefore our adaptive hash algorithm generator dynamically generates hash functions in terms of a set of input pairs 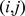 in which 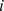 indicates which word of the window to select, and 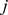 specifies a bit shift to apply to that word (positive and negative values indicating right and left shifts, respectively). The hash itself is computed by XORing the result of 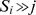 (or 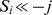 if 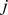 is negative) of all 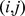 input pairs. This process is illustrated with a practical example in Figure 4.

These hash functions themselves are simple and fast to compute; however the number of possible hash functions is extreme. For any given spaced seed pattern of length 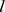, there are 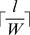 choices of word, and 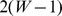 choices of shifts for each hash input pair, and therefore 
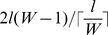
 possible pairs. Because input pairs are combined by XOR, applying the same input twice is equivalent to not applying it at all. Therefore there are exactly 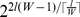 or 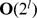 possible hash functions. The vast majority of these hash functions are undesirable as they use an excessive number of inputs or leave some parts of the hash underutilized. Therefore finding the “good” hash functions is a nontrivial problem. Our adaptive hash generator solves this problem by using a genetic algorithm to iteratively explore the space of possible hash functions. In this approach, we create a population of (initially random) hash functions, and each cycle they are evaluated for “fitness” based on their expected entropy and computation cost (method described in Materials S1). The highest scoring third of hash functions are randomly combined and mutated to generate new hash functions, and the lowest scoring third are eliminated. By default we start with 100 hash functions, and repeat for at least 1000 cycles or until the marginal improvement of the best hash function drops below a given threshold.

## Results

### Experiment Design

Because Murasaki focuses solely on multisequence anchor identification, it is difficult to identify a “drop-in replacement” from existing toolchains against which to compare Murasaki. Murasaki has already been used in several projects including orthologous segment mapping [21], and a study of *Pseudomonas aeruginosa* that revealed the occurrences of large inversions in various *P. aeruginosa* chromosomes [29]; so it is known empirically to be a useful tool. To quantitatively test accuracy and efficiency of Murasaki we evaluated Murasaki's performance under several controlled scenarios with respect to speed and accuracy. Our tests focus on either whole genomes, or when the whole genome would be cost-prohibitive, just the X chromosome for expediency. The concerns that we address in our testing include:

1. Comparison to existing methods
2. Adaptive hash algorithm performance
3. Parallelization and scalability in cluster-computing environments
4. Performance on large inputs

Lacking a perfect drop-in replacement for an existing method, we chose to work with BLASTZ [10] to generate pairwise anchors and TBA [22] to combine BLASTZ's anchors into multisequence anchors when needed. BLASTZ is widely used as another Swiss-army knife of homology search, and provides options to return anchors at the ungapped-alignment stage similar to Murasaki. We cannot force BLASTZ to use longer spaced seed patterns, and recognize this is not BLASTZ's intended use, but it can be made to fulfill the same basic anchor finding functions. The combination of BLASTZ with TBA is consistent with the intended use of TBA. We use blastz.v7 with options “C = 3 T = 4 M = 100 K = 6000” to run BLASTZ with pattern settings similar to Murasaki. The “C = 3” parameter skips the gapped extension and chaining steps, outputting only HSPs (“high scoring pairs”), effectively anchors just like those of Murasaki. The “K = 6000” score threshold was selected based on existing studies using BLASTZ on mammalian genomes [10]. For TBA, we used tba.v12 with default parameters and TBA's all_bz program to run BLASTZ with the above specifications. Murasaki's parameters, primarily “–scorefilter = 6000” and “–mergefilter = 100”, approximate the BLASTZ settings. “Mergefilter” prevents generating anchors from seeds which would incur more than the specified number of anchors, tagging these regions as “repeats”. Additionally repeat masked sequences [30] were obtained from the Ensembl genome database [31]. Although the Murasaki “mergefilter” option provides some robustness against repeats, for mammalian genomes using repeat masked sequences reduces the amount of sequence that must be hashed and stored in memory by approximately one half (see Materials S1).

### Comparison to existing methods

We applied both Murasaki and the BLASTZ+TBA approach described above to the X chromosomes of eight mammals: human, mouse, rat, chimp, rhesus, orangutan, dog, and cow. We compared every combinatorial choice of two species, then every choice of three species, and so on. For the final case of eight species, we repeated the test five times to account for variability in computation time. For Murasaki we used the 24 base spaced seed pattern 101111110101110111110011 to have a pattern close to the BLASTZ level of sensitivity (the method used to choose that pattern is explained in Materials S1).

First we show that Murasaki and TBA have comparable accuracy. Because Murasaki is using different spaced seeds than BLASTZ and requires that seeds match in *all* input sequences (unlike TBA where less similar matches are introduced during progressive multiple alignment), a direct comparison of individual anchors between Murasaki and TBA is not helpful as we would not expect them to find the same anchors. However, we would expect that both Murasaki and TBA should accurately anchor areas of significant similarity such as orthologous genes, and that both Murasaki and TBA would find anchors in same vicinity regardless of gene content (i.e., anchoring the same orthologous segments). We use those two ideas as the basis for our comparison.

**Figure 5:**
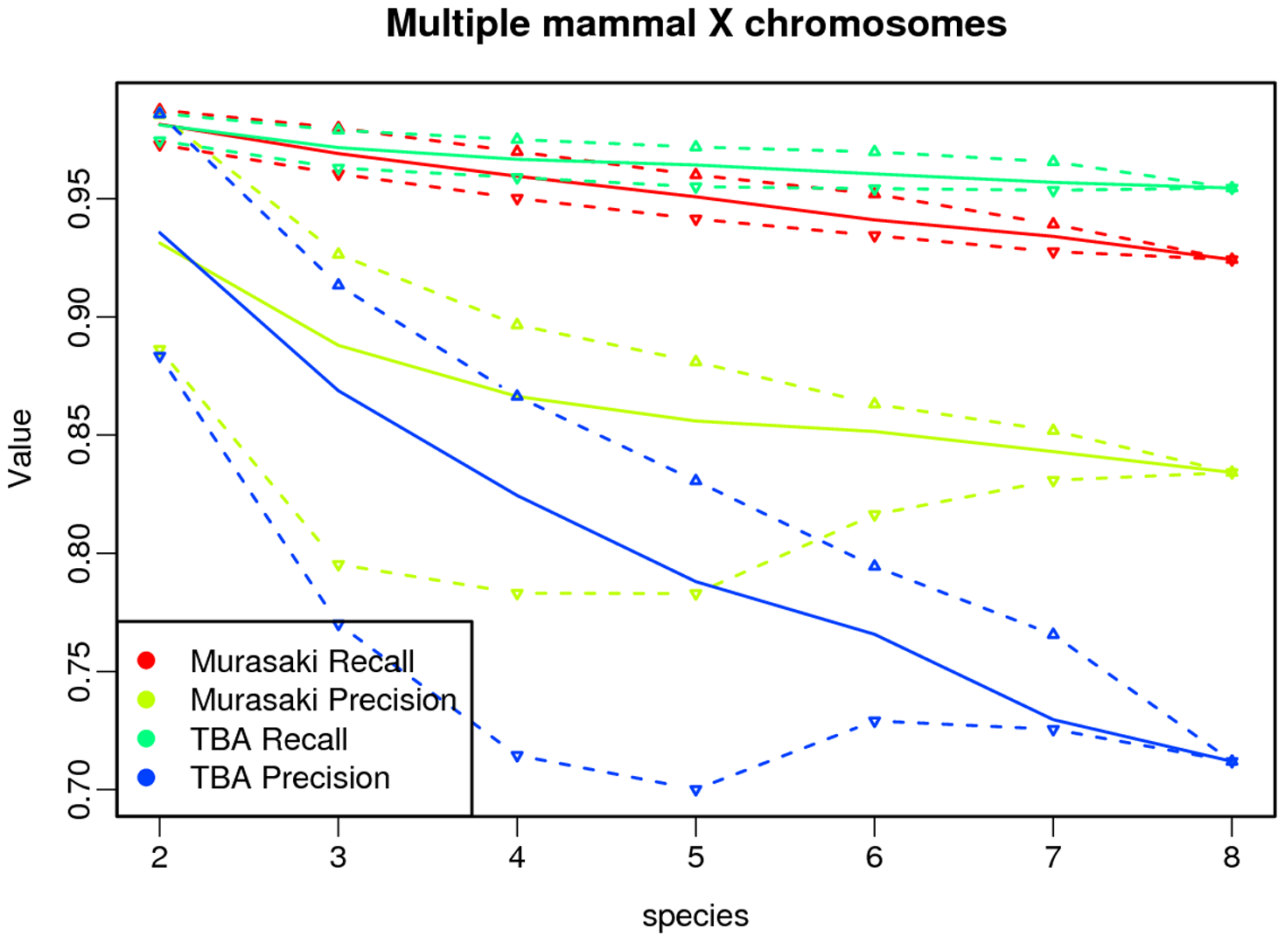
Ortholog consistency in multiple genome comparison. This graph examines the consistency of anchors with known orthologs when comparing varying numbers of multiple mammalian X chromosomes (from 2 to 8 different species), using both Murasaki and TBA. Consistency is evaluated in terms of recall (the percent of known orthologs anchored), and precision (the percent of anchors incident on known orthologs to correctly include the other known orthologous set members). The solid line represents the median of all tests for that number of species, while the dashed lines represent the first and third quartiles. In this graph it can be seen that as the number of species increases, both precision and recall decline with both Murasaki and TBA, however Murasaki's precision remains significantly higher than TBA.

First, we evaluated the precision and recall of Murasaki and TBA in terms of anchors found in orthologous genes. Sets of orthologous genes were used as defined in [21] and retrieved from the SPEED ortholog database [32]. Here “recall” and “precision” are defined in terms of “consistency” such that each anchor overlapping a known ortholog is classified as either “consistent” or “inconsistent,” and anchors not overlapping any known ortholog are neither. An anchor is counted as “consistent” if and only if it overlaps each member of the orthologous gene set. Likewise an anchor is “inconsistent” if and only if it overlaps at least one member of an orthologous gene set but fails to overlap at least one of the other orthologous genes. “Recall” is then defined as the ratio of orthologous gene sets correctly detected by at least one consistent anchor compared with the total number of orthologous gene sets. “Precision” is defined as the ratio of consistent anchors to the number of anchors either consistent or inconsistent. As a combined overall score, we compute the F-score which is defined as the harmonic mean of precision (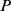) and recall (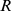):
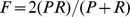



As shown in Figure 5, both Murasaki and TBA are similar terms of both precision and recall. For Murasaki, that recall drops off as more sequences are added; however, precision increases significantly. The same trend is visible in TBA; however, the effect is more pronounced in Murasaki where the increase in precision is far more significant, resulting in a significantly higher overall F-score, as shown in Table 1. The primary reason for this difference in performance characteristics is that Murasaki anchors are calculated across multiple genomes simultaneously rather than progressively, decreasing the number of erroneous matches at the cost of some sensitivity.

**Table 1:** Multiple X chromosome test results.

	BLASTZ+TBA				Murasaki			
Species	Time (s)	Recall	Precision	F-Score	Time (s)	Recall	Precision	F-Score
2	154	0.981	0.936	0.956	349	0.981	0.931	0.954
3	459	0.972	0.869	0.916	457	0.969	0.888	0.924
4	906	0.967	0.824	0.890	587	0.960	0.866	0.910
5	1489	0.964	0.788	0.869	806	0.951	0.856	0.899
6	2276	0.961	0.766	0.852	961	0.941	0.852	0.892
7	3215	0.957	0.730	0.828	1133	0.934	0.843	0.889
8	4437	0.955	0.712	0.816	1516	0.924	0.834	0.877

This table shows the median statistics from the multiple X chromosome test. Median total computation time, recall, precision, and F-score are shown for each number of species compared using both Murasaki and BLASTZ+TBA. The BLASTZ+TBA computation time includes only the BLASTZ portion of the calculations.

Second, we used the anchors produced by each algorithm to predict orthologous segments. Orthologous segments refer to an uninterrupted region of collinear homology between several genomes; that is segments unlikely to have undergone genomic rearrangement from their common ancestor [21]. There are a number of algorithms for identifying orthologous segments, the most simple of which is GRIMM-Synteny [33] where anchors at distances less than a user-specified threshold are merged into “syntenic blocks.” In this study, we chose to use OSfinder [21] because it uses Markov chain models to find optimal parameters by maximizing the likelihood of the input dataset. This approach provides an anchor algorithm agnostic means to predict orthologous segments using anchors from either Murasaki or TBA.

**Figure 6:**
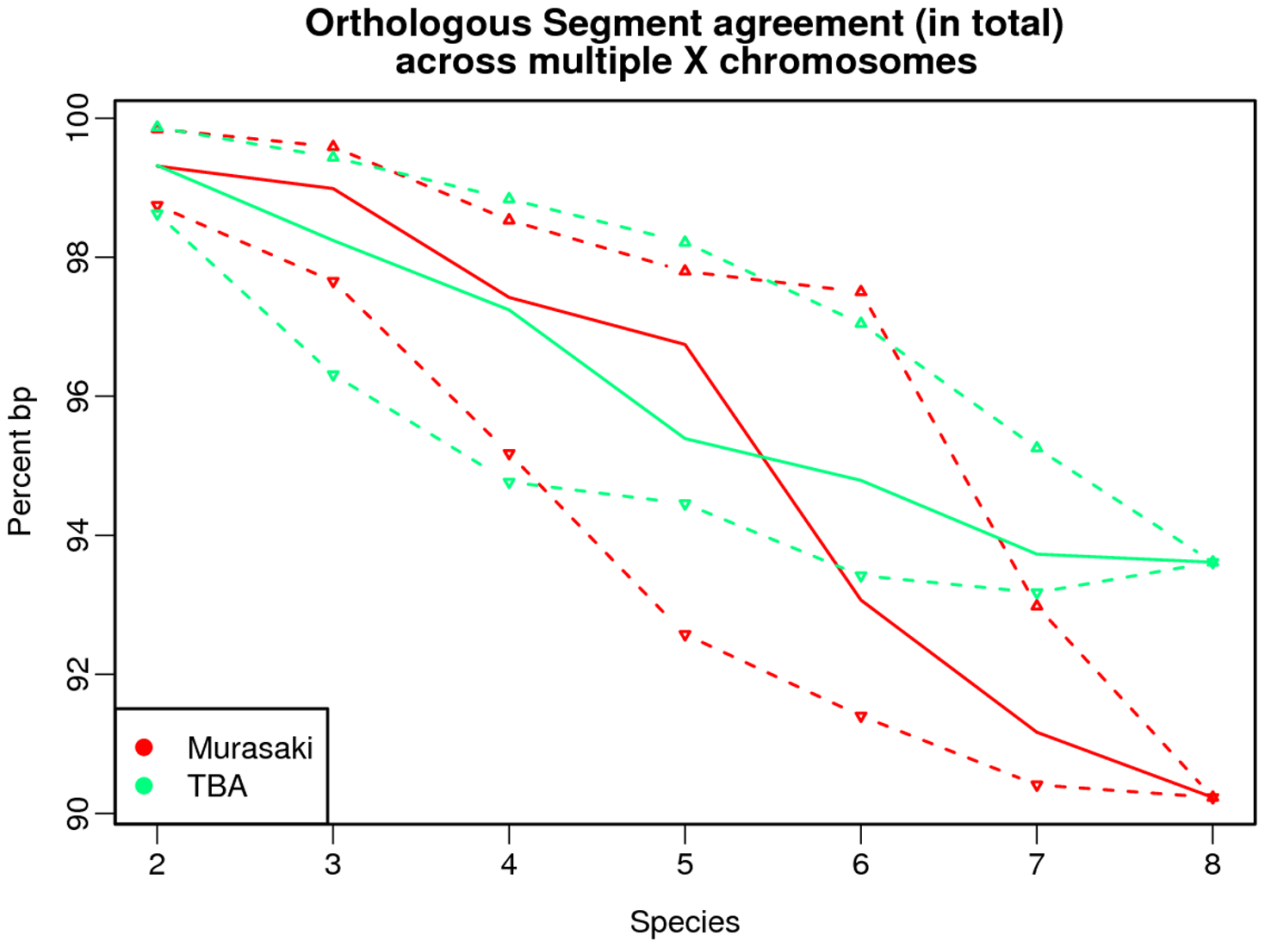
Orthologous segment agreement across multiple X chromosomes. This graph shows the result of comparing othologous segments as identified by OSfinder using anchors from Murasaki and TBA. The quantities shown here are the percent of base pairs in each orthologous segment shared by the other. The solid line represents the median of all tests for that number of species, while the dashed lines represent the first and third quartiles. For example, with anchors generated from all 8 species, 94% of the base pairs in the orthologous segments generated from TBA's anchors were also identified as part of an overlapping orthologous segment by anchors from Murasaki.

**Figure 7:**
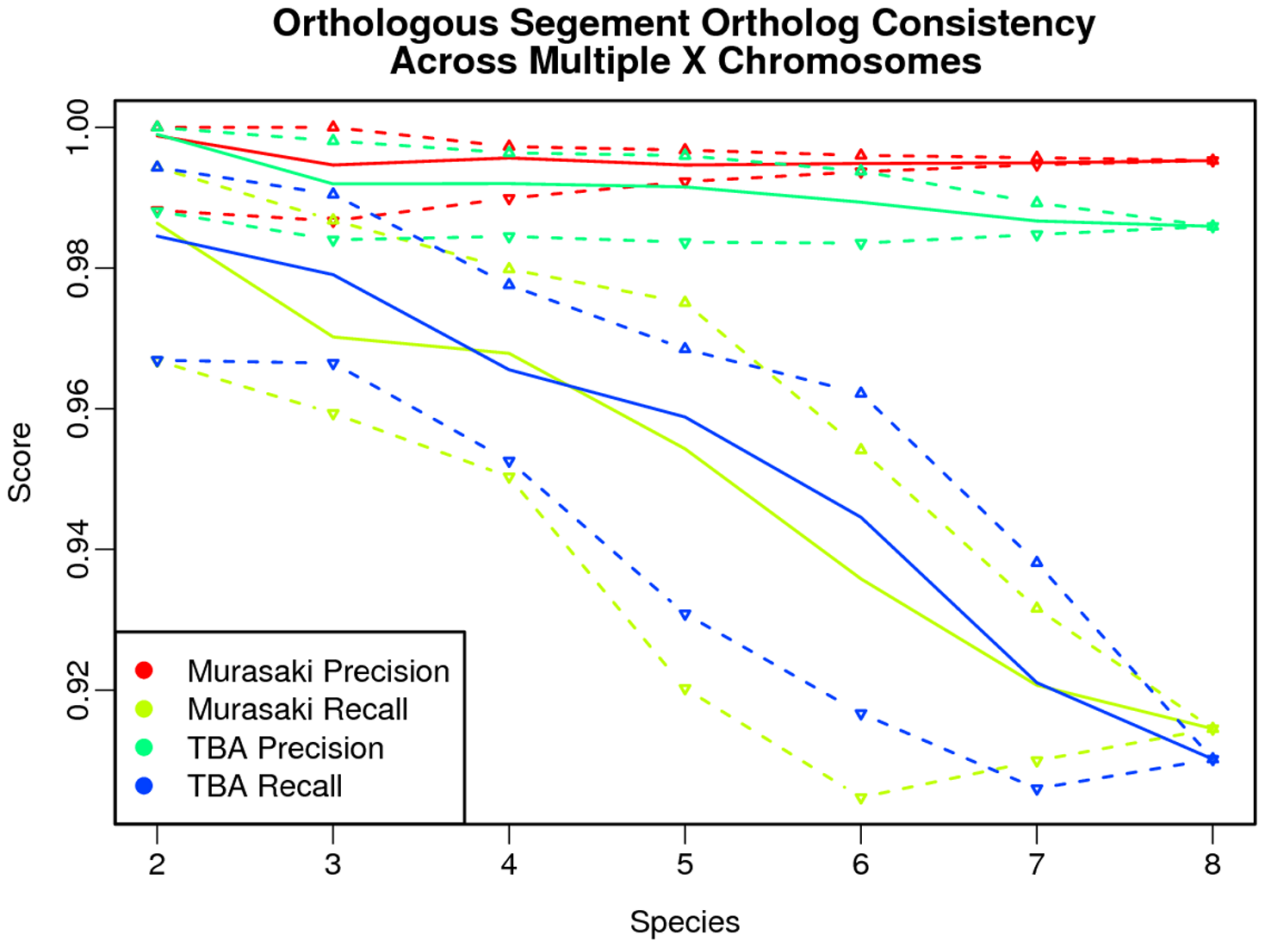
Orthologous Segment Ortholog Consistency Across Multiple X Chromosomes. This graph shows the result of evaluating the orthologous segments returned produced by anchors from Murasaki and TBA with OSfinder. The solid line represents the median of all tests for that number of species, while the dashed lines represent the first and third quartiles. The precision and recall are calculated as described in Comparison to existing methods. The orthologous segments produced using both TBA and Murasaki are nearly identical in terms of recall, however Murasaki outperforms TBA in terms of precision in our comparisons of large numbers of species. We note however that both Murasaki and TBA perform very well with all precision and recall scores above 90%.

We compared the orthologous segments from OSfinder in terms of the extent to which the resulting orthologous segments overlap as measured in base pairs, and again in terms of orthologous gene recall and precision as confirmation. As shown in Figure 6, the orthologous segments detected via both Murasaki and TBA share over 90% of the same bases for multiple alignments and, and over 99% at in pair-wise comparisons. To confirm that OSfinder's orthologous segments are accurate when using either algorithm, we evaluated the orthologous segments as before in terms of consistency with known orthologous genes. In terms of orthologous gene consistency, there was no significant difference between orthologous segments using Murasaki and TBA as shown in Figure 7.

**Figure 8:**
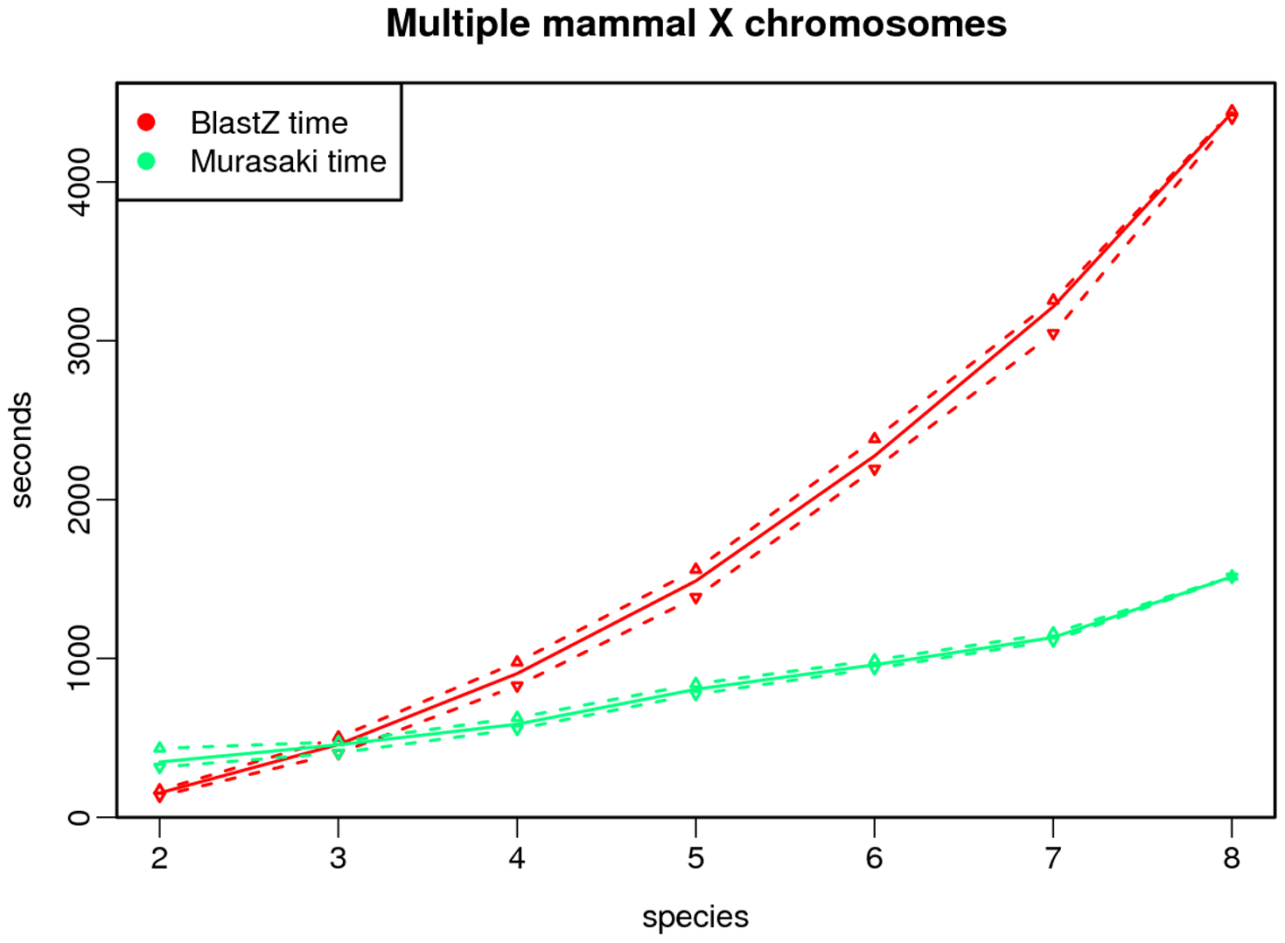
Computation time for multiple mammalian X chromosomes. This graph compares the computational time required to compare multiple X mammalian X chromosomes using Murasaki and the BLASTZ component of TBA. Because TBA requires all pairwise comparisons of the genomes under alignment, the time required for TBA grows quadratically, while Murasaki's time is near linear. The solid line represents the median of all tests for that number of species, while the dashed lines represent the first and third quartiles.

Finally we compare computation time. We are only concerned about time spent on anchor computation. Because in TBA we cannot separate its time spent generating progressive alignments from time spent generating multigenome anchors, we ignore the computation time from TBA and report only BLASTZ time. Consequently this slightly underreports the actual time required to generate multigenome anchors using BLASTZ and TBA, but if Murasaki is faster than the BLASTZ computation portion alone, then it is necessarily faster than BLASTZ and TBA combined; therefore, this comparison is sufficient for our purposes. The resulting computation times are shown in Figure 8. For pair-wise comparisons, BLASTZ is faster; however, when anchoring three or more sequences, Murasaki is significantly faster than BLASTZ. Because using TBA requires each pair-wise comparison, the computation time increases quadratically with each additional sequence under comparison. On the other hand, Murasaki's computation time increases at approximately an 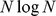 rate (however for these cases with only two to eight mammalian genomes, only the linear 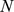 term is apparent). This is because all matching seeds are found simultaneously after being entered in the hash table together; therefore because Murasaki's runtime is bounded by the total input length 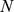, not sequence number. The difference between Murasaki and pair-wise methods increases dramatically as the number of sequences increases. The computation times for these tests are shown in Table 1.

### Adaptive hash algorithm performance

To evaluate the performance of our adaptive hash algorithm, we compared it with the standard cryptographic SHA-1 and MD5 hash algorithms, and the First-N approach. Being designed for cryptographic use, we expect SHA-1 and MD5 hash algorithms to provide near-random utilization of the key space while being more computationally expensive. To test this, we ran Murasaki on Human and Mouse X chromosomes using five different patterns, over different hashbits settings, repeating each trial four times. We then compared the number of unique keys produced by each hash function to the median number of keys produced by the adaptive hash algorithm, as shown in Table 2. Our adaptive hash algorithm performs within %0.05 of the cryptographic hash functions, while the naive First-N approach lags 32% behind any of the others. We find that as hash keys are used, fewer collisions require less work to invert the hash table, resulting in faster extraction times, as shown in Table 2. Table 2 also shows the computational time required to hash the input sequences under each hash function. As expected, the cryptographic functions were significantly (between 52% and 80%) slower. It is worth noting that hash computation times required by our naive First-N hash function exceeded even the cryptographic MD5 and SHA-1 hash functions. Even though calculation of our naive First-N hash function is conceptually extremely simple, it is computationally inefficient compared to hash functions that incorporate spaces using the pattern optimized approach used in the adaptive and cryptographic hash functions. The combined effect of hash time and extraction time is apparent in Table 2, showing the total processing time required using each hash function. Overall run-time using the adaptive hasher was 15% to 20% faster than the cryptographic hashers, and 23% to 30% faster than the naive approach. Percentages of key utilization, and times for extracting and hashing are shown relative to our adaptive hasher in Figures S1, S2, and S3.

**Table 2:** Total computational time by hash algorithm and hashbits.

	Hash algorithm			
Statistic measured	Adaptive	MD5	SHA-1	First-N
Hash Time (s)	124.908	188.449	208.954	218.202
Extract Time (s)	200.554	197.254	196.82	218.334
Total Time (s)	325.798	386.706	405.385	437.65
Hash keys used	1	1.00018	1.00018	0.71606

This table shows the median total computational time, along with separate times to hash and extract anchors required by different hash algorithms when anchoring human and mouse X chromosomes. The final line shows the median number of hash keys used by each hash algorithm relative to the number used by the Adaptive hash algorithm.

**Figure 9:**
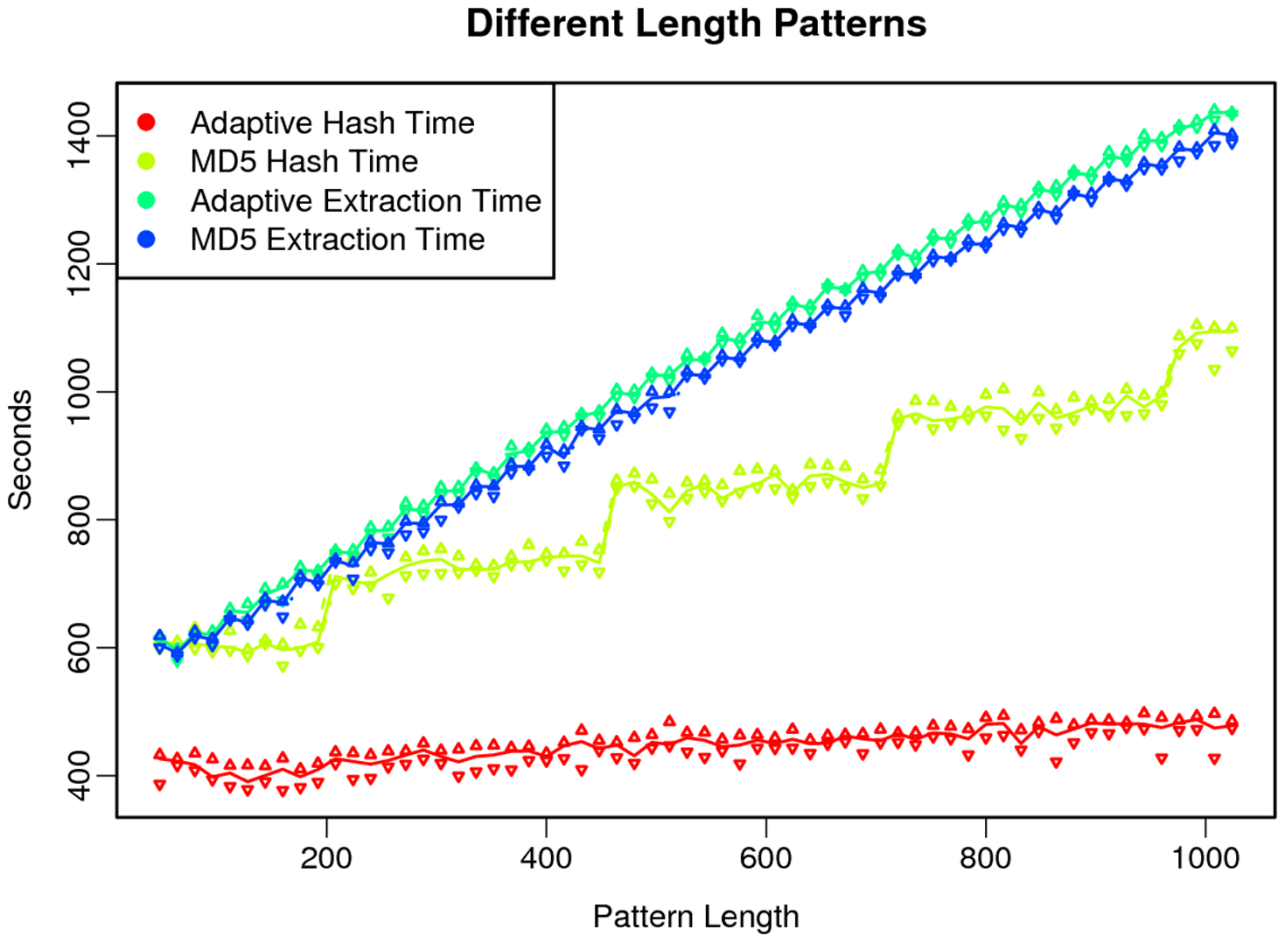
Hash and extraction times using Adaptive and MD5 hash algorithms with very long patterns. This graph shows the hash and extract computation time required to compare human and mouse X chromosomes using very long patterns, and the difference between MD5 and Adaptive hash algorithms. The difference between MD5 and Adaptive in hash time grows significantly with pattern length, whereas the difference in extraction time is minuscule compared with the overall time required.

We also tested Murasaki on Human and Mouse X chromosomes using different random patterns of lengths from 48 to 1024 at multiples of 16. Five random patterns were generated for each length, and each pattern had a weight 75% of its length. Each test was repeated three times to reduce the variability in timing. As shown in Figure 9, the adaptive hash functions consistently outperformed MD5 in hashing time while maintaining an extraction time almost identical to MD5. The stair-step appearance of the hash times of MD5 is due to the way that MD5 processes input in blocks, and when input lengths roll over such a block boundary, a new round of calculations is incurred. In contrast the hash time for the adaptive hash algorithm grows very slowly with regard to pattern length, because estimation of the expected hash function entropy allows Murasaki to predict the point at which adding additional inputs no longer provides significant gains for the current hash key size. The percentages of keyspace used with these long patterns is shown in Figure S4.

### Scalability in cluster-computing environments

Based on the parallel algorithm design, we expect the peak efficiency of the parallel computation to vary depending on the interconnect speed of the nodes; however because the computationally intensive tasks can be split into independent sets and divided evenly between nodes, we expect the execution time to decrease by a multiple of the number of active nodes. In other words, for 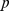 processors and a given input, where Murasaki finish in time 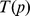, we expect the speedup 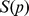 to grow linearly with 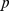 as in 
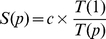
 for some constant 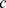.

To test Murasaki against this hypothetical performance, we used Murasaki to anchor Human and Mouse chromosomes using between 2 and 40 processors across 10 machines. We used OpenMPI on Torque as our MPI implementation, and each CPU was a dual core Opteron 2220 SE, with two CPUs per machine (ie. 4 cores per machine) and had between 16GB and 32GB of RAM available. In fact, because the amount of RAM available for use as a hash table grows with the number of machines used, the actual speed-up may be greater than linear for large inputs and large numbers of processor elements. To test the scalability of Murasaki on large inputs, we ran our tests using and the whole human and mouse genomes across the largest number of CPUs we had available.

**Figure 10:**
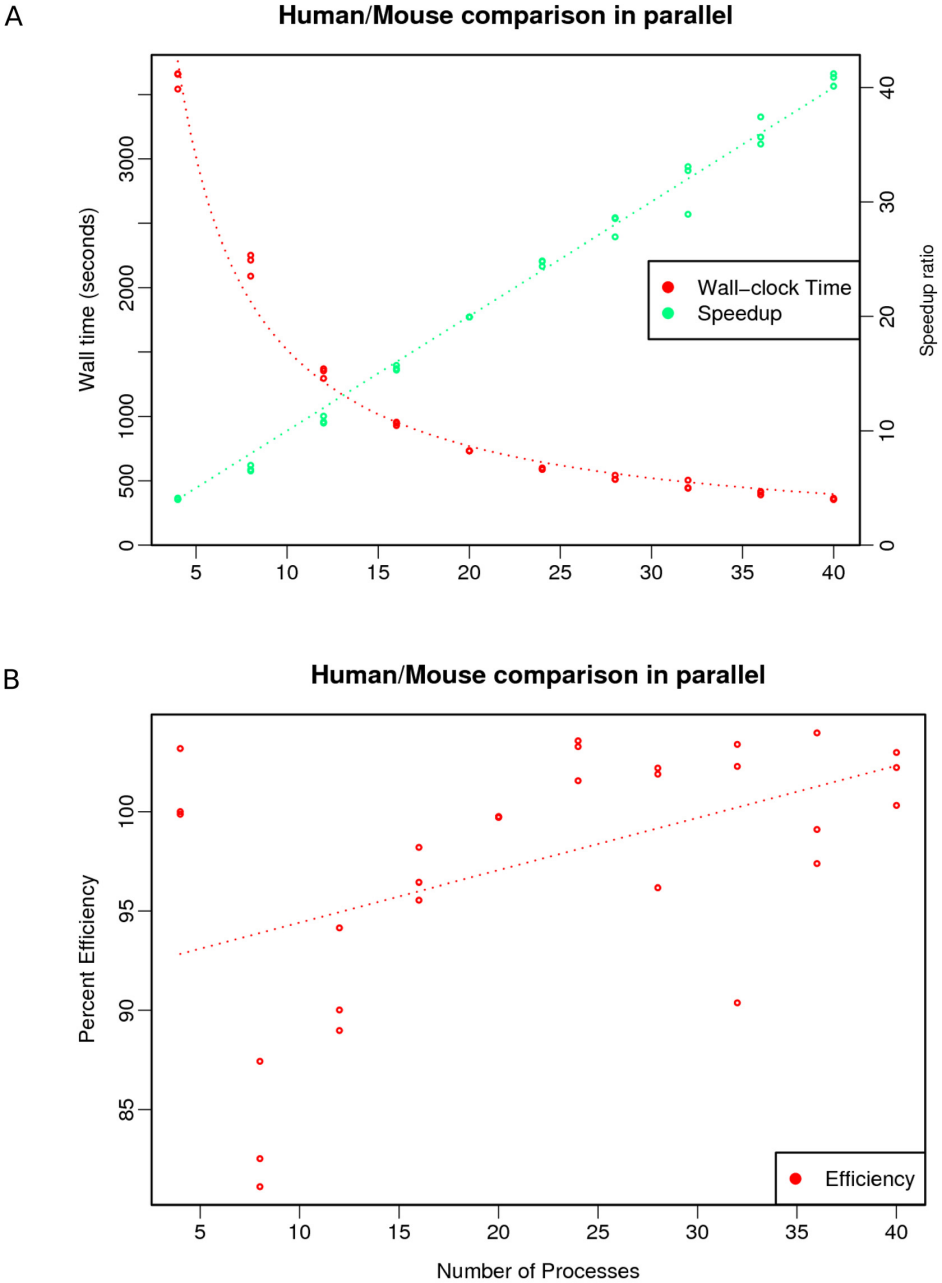
Parallel computational wall time, speedup, and efficiency of complete human and mouse genomes comparisons. These graphs illustrate the computational time required for a comparison of human and mouse genomes using different numbers of processors. In **A** the wall clock time is shown in red using the left axis with the corresponding “speedup” (
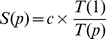
) shown in green using the right axis. Least-squares regression lines have been fitted to each dataset, highlighting the near perfectly linear speedup and inversely decreasing wall clock times. In **B** the parallel computation “efficiency” (
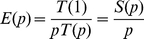
) is reported. Again a least-squares regression line is fitted to the data. Here the increasing least-squares regression line highlights the fact that on average the increase in speedup is greater than would be expected if 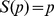.

Figure 10A shows the resulting decrease in wall clock time required as the number of processors increases, and the coressponding speedup value. Because the whole genome comparison requires too much memory for any single machine in our cluster, 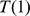 is estimated to be 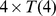. We have fitted least-squares linear regression lines to each set of values, and found the speedup constant 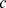 to be 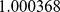 with 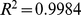. While the 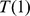 value is available only as an estimate, the close fit to a linear model shows that the algorithm scales favorably. Critically the parallel efficiency (
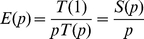
) shown in Figure 10B appears to increase with respect to the number processors, the most desirable yet elusive pattern in parallel algorithms. This increase in efficiency is due in part to the increasing hash table size; however all tests with 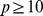 have access to all the machines' memory and utlize the same 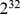 entry hash table, therefore we speculate that the remaining increase likely relates to improved scheduling and cache performance as each nodes' work becomes increasingly localized.

### Performance on large inputs

**Figure 11:**
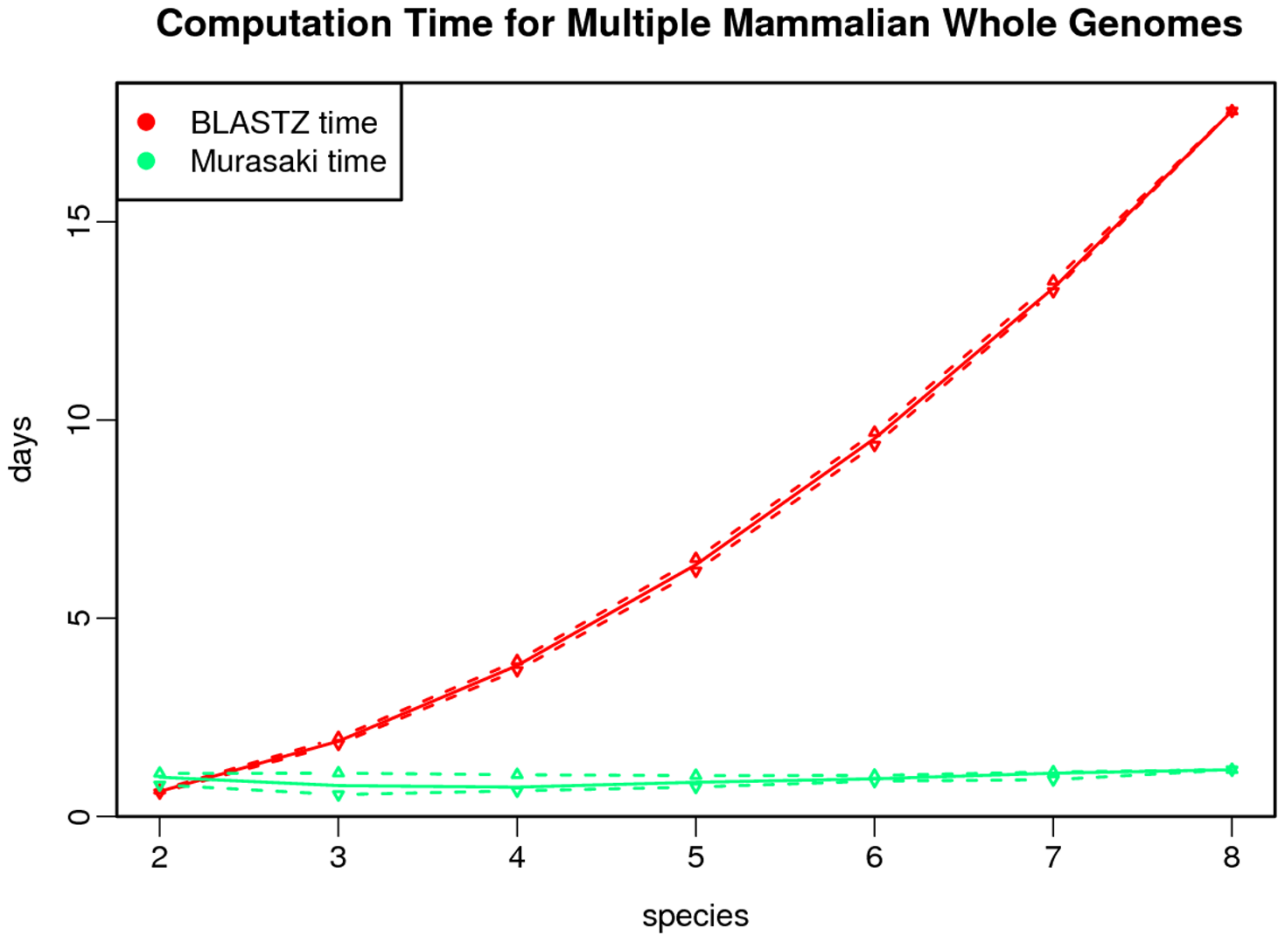
Computational time required to anchor multiple mammalian whole genomes. This graph shows the median CPU time in days required to anchor different numbers of mammalian whole genomes using TBA and Murasaki. The times for TBA include only the time spent on pairwise BLASTZ comparisons. The solid line represents the median of all tests for that number of species, while the dashed lines represent the first and third quartiles.

To test the scalability of Murasaki for full multiple genome comparisons, we repeated the comparison to existing methods test on eight mammals (human, mouse, rat, chimp, rhesus, orangutan, dog, and cow), however this time using the whole genome rather than just the X chromosomes. Again, for BLASTZ and TBA we measure only the computational of BLASTZ alone. We used the same pattern and other settings as before; however, this time we ran Murasaki in parallel across 10 machines using 40 cores as in the scalability test above, using a fix hash bits setting of 29. We report the total median CPU time used by Murasaki and BLASTZ along with recall, precision, and F-score statistics in Table 3 for all combinations for each number of sequences. The scalability cost of the BLASTZ+TBA combination is even more striking in this case as BLASTZ is unable to compare input whole genomes, requiring the user to compare each chromosome combination (Human-1 and Chimp-1, Human-1 and Chimp-2, etc.) for each species combination (Human and Chimp, Human and Rhesus, etc.). Consequently the resulting graph of these times shown in Figure 11 makes Murasaki appear nearly constant by comparison to BLASTZ. When evaluated against gene orthology dataset as in the test cases above, the overall first, second, and third quartile F-Scores from all combinations of these whole genomes are 0.832, 0.861, and 0.896 respectively, leading us to believe that these anchors are approximately as accurate as those found in the X chromosome tests above.

**Table 3:** Mammal whole genome comparisons.

Species	TBA-BLASTZ CPU Time (days)	Murasaki CPU Time (days)	Recall	Precision	F-Score
2	0.632	0.991	0.971	0.910	0.925
3	1.901	0.780	0.953	0.863	0.900
4	3.808	0.741	0.922	0.832	0.867
5	6.351	0.864	0.887	0.807	0.840
6	9.534	0.951	0.855	0.790	0.818
7	13.328	1.093	0.824	0.768	0.797
8	17.796	1.180	0.790	0.764	0.777

This table shows median computation times and accuracy for mammal whole genome comparisons with respect to each number of species under comparison. Recall, precision, and F-Score were calculated from Murasaki anchors only.

**Figure 12:**
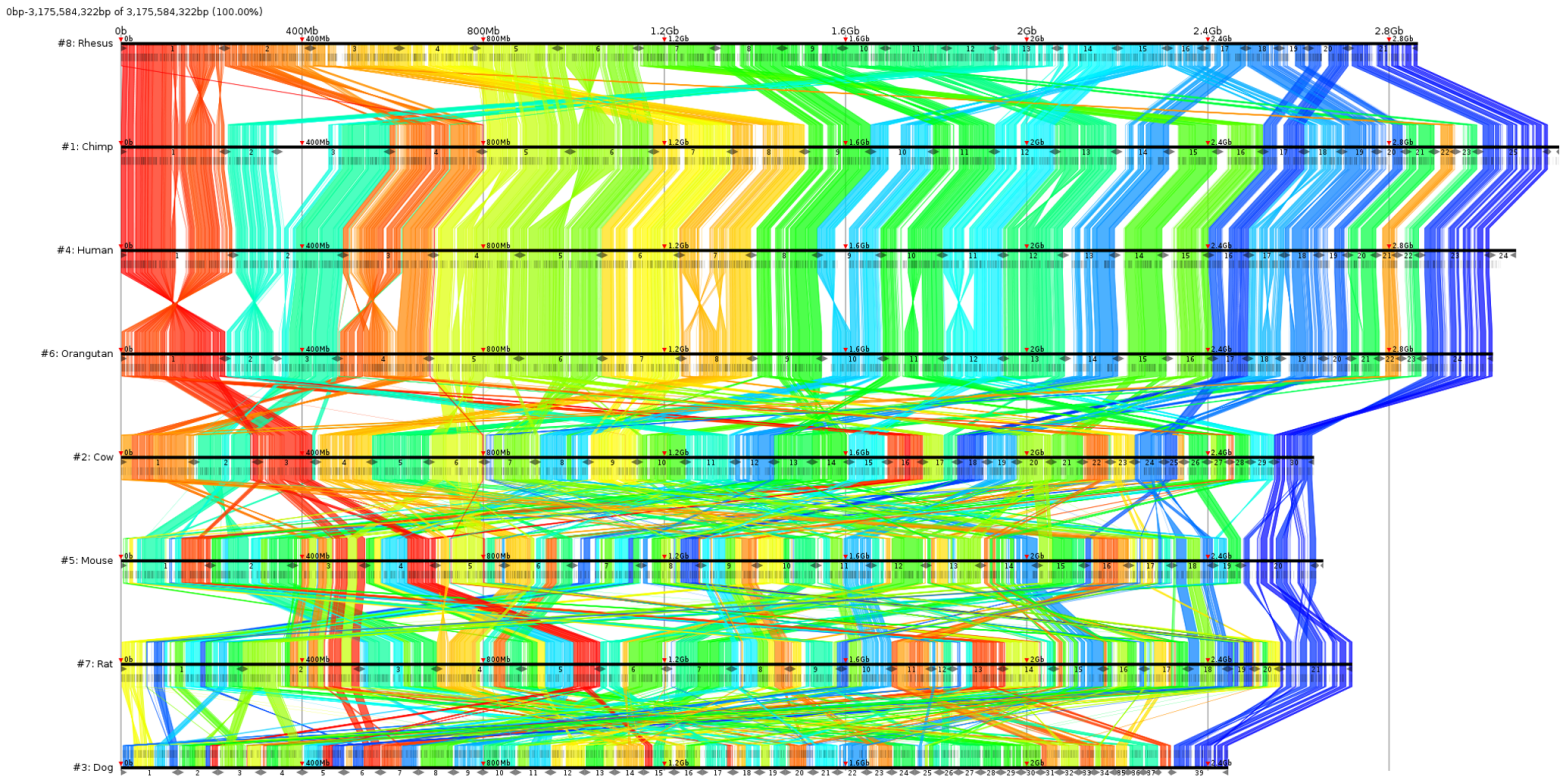
Anchors between 8 mammalian whole genomes. This figure shows the resulting anchors from our comparison of 8 mammalian genomes (from top to bottom): rhesus, chimp, human, orangutan, cow, mouse, rat, and dog. Anchors are drawn as colored lines from one sequence to the next. The color is determined by the anchor's position in the first (rhesus) genome, making it easier to see rearrangements and where the other genomes are related. Chromosomes are denoted by the number shown between ▸ and ◂ symbols along each genome. The sex chromosomes are shown at the right end (e.g., 23 (X) and 24 (Y) for human).

The eight species comparison anchors (drawn using GMV [34]) are shown in Figure 12. All of these comparisons are available for download and interactive browsing with GMV [34] from the Murasaki website (http://murasaki.dna.bio.keio.ac.jp).

## Discussion

### Choice of comparison algorithm

Because BLASTZ is optimized for pair-wise comparisons, it can be expected to do well on a small number of inputs. However, because all-by-all comparisons are required to generate multiple alignments, the time required is expected to grow quadratically with the number of input sequences. In contrast, Murasaki is designed to compare an arbitrarily large number of genomes simultaneously, and assuming a linearly bounded number of anchors, the computation time for Murasaki is expected to be approximately 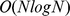 for a total input length of 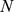.

It might then seem that rather than BLASTZ, a better comparison of Murasaki would be to a natively “multiple” alignment program like Mauve; however it is important to note that Murasaki performs a fundamentally different function than Mauve in that Mauve aligns whole collinear regions bounded by *unique* anchors. While these anchors are in some ways analogous to Murasaki's anchors, the requirement of “unique anchors” puts Mauve in a fundamentally different arena, where its strength lies in alignment rather than anchoring. Also, while Mauve is well suited to bacterial genomes, it is not well suited for mammalian scale genomes (it is reportedly not impossible [4], but this use is not recommended, it does not work without applying some undocumented options to perform the necessary out of core sort, and we could not replicate or verify the results).

We also tested another alternative from the TBA package called Roast which appears to implement the method described in [23] which builds a multiple alignment based on pairwise comparisons between a reference sequence and all other sequences, thus in theory requiring time linear with respect to the number of sequences, similar to Murasaki. However due to an apparent bug in the implementation, Roast actually is actually worse than TBA in some cases. Even assuming that the were fixed, however, the fragmentation required to compare sequences via BLASTZ results in time requirements which grow several times faster than Murasaki at whole genome scales. The results from our fixed version of Roast and the native Roast comparison are included and discussed in Materials S1 (see Figures S5 and S6).

### Bottlenecks in parallelization

Under the parallel algorithm, when hasher nodes send seeds to storage nodes, the choice of storage node is determined by the hash key. This means that balance and contention between storage nodes is, ultimately, determined by the input sequences. For example a sequence containing only one type of base (e.g., 4 Gbp of AAAAA) would necessarily all get sent to the same storage node, causing a less than optimal distribution of storage and heavy contention for that node. This is in fact the worst case, and highly improbable with real-world genomes, but similar factors can unbalance the load between storage nodes. This problem is mitigated by the near uniform random output of Murasaki's hash functions, making it approximately equally unlikely that any two given seeds share the same node, but it does not help the worst case. A modification to the hash table data structure might allow storage nodes to dynamically update their active hash table region, and redirect overexpressed seeds to less heavily loaded storage nodes. This would of course require some additional overhead.

### Parallel overhead

While adding machines to a cluster can increase the amount of available RAM indefinitely, the storage of the input sequences themselves in memory incurs a constant cost per machine added. Shared memory is used to mitigate this cost by loading only one copy per machine (rather than per processor), and input sequences are stored 2 bits per bp. However, as the size of the input sequence grows, at some point merely loading all the sequences into memory exhausts the system's memory. Thus the smallest memory machine in the network effectively limits the maximum input size of Murasaki. For example loading all 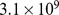 bp of the human genome takes about 738MB. Comparing ten mammals requires at least 7GB per machine, and that is not including any space for the hash table.

### Conclusions

We have shown that our anchoring algorithm Murasaki produces accurate anchors across multiple genomes with a computational efficiency significantly greater than existing methods. Its adaptive hash function generation algorithm provides an efficient method to use arbitrary spaced seeds of any length with collision rates close to pseudorandom one-way cryptographic hash algorithms at a fraction of the computational cost. Additionally, our method is highly scalable, allowing whole computer clusters to be fully utilized for large-scale multiple genome comparison.

We have shown that our anchoring algorithm Murasaki produces accurate anchors across multiple genomes with a computational efficiency significantly greater than existing methods. Its adaptive hash function generation algorithm provides an efficient method to use arbitrary spaced seeds of any length with collision rates close to pseudorandom one-way cryptographic hash algorithms at a fraction of the computational cost. Additionally, our method is highly scalable, allowing whole computer clusters to be fully utilized for large-scale multiple genome comparison.

### Availability and requirements

Project name: Murasaki

Project home page: http://murasaki.sourceforge.net

Operating system(s): Any POSIX compatible OS (e.g., Linux, FreeBSD, MacOS X).

Programming language: C++ and Perl.

Other requirements: Boost and zlib libraries.

License: GPLv3.

## Supporting Information

Figure S1 Hash keys used in comparison by SHA-1/MD5 hash algorithms in comparison to adaptive hashing at different hashbit values. This graph doesn't include First-N in order to examine, and adaptive hash results to examine the minute difference between Adaptive, SHA-1, and MD5. Only for large hash keys (high values of hashbits) does adaptive diverge significantly from SHA-1 and MD5, and even then the difference is minuscule.
(0.05 MB TIF)

Figure S2 Extract time required by each hash algorithm compared to the adaptive hash algorithm. This graph shows the relative time required to extract matching seed sets from the hash table under different hash functions compared to the median time required our adaptive hash function. The solid line shows the median of all trials, while the dashed lines show the first and third quartiles.
(0.06 MB TIF)

Figure S3 Time required to hash human and mouse X chromosomes using different hash functions at various hashbits settings compared to Adaptive. Here, we examine the difference in time required to compute hashes, store each *(K,V)* pair at different hashbits settings, again compared to our adaptive hash method. It's interesting to note that the naive First-N approach performs more poorly than even the slowest cryptographic hasher.
(0.05 MB TIF)

Figure S4 Comparing keyspace usage of Adaptive and MD5 hash functions for very long patterns. This graph shows the percent of possible hash keys produced by Adaptive and MD5 hash functions when hashing human and mouse X chromosomes. The number of hash keys possible increases with pattern length, because the number of observed unique seeds increases. Our adaptive hash algorithm keeps up with MD5 even for extremely long patterns.
(0.05 MB TIF)

Figure S5 Computational time required to anchor multiple mammalian whole genomes. This graph shows the median CPU time in days required to anchor different numbers of mammalian whole genomes using TBA, Murasaki, and the patched and unpatched versions of Roast. The times for TBA and Roast include only the time spent on pairwise BLASTZ comparisons. The solid line represents the median of all tests for that number of species, while the dashed lines represent the first and third quartiles.
(0.05 MB TIF)

Figure S6 Computation time for multiple mammalian X chromosomes. This graph compares the computational time required to compare multiple X mammalian X chromosomes using Murasaki and the BLASTZ components of TBA, Roast, and our patched version of Roast. Because TBA requires all pairwise comparisons of the genomes under alignment, the time required for TBA grows quadratically, while Murasaki's time is nearly linear. The solid line represents the median of all tests for that number of species, while the dashed lines represent the first and third quartiles.
(0.06 MB TIF)

Materials S1 Additional detail on the implementation of hash functions, data structures, hash function fitness evaluation, pattern selection, runtime parameters, and our comparison with Roast.
(0.39 MB PDF)
