# Supplementary figures and images for: Murasaki: A Fast, Parallelizable Algorithm to Find Anchors from Multiple Genomes
Source: PLoS One. 2010 Sep 24;5(9):e12651. doi: 10.1371/journal.pone.0012651 (PMC2945767; doi:10.1371/journal.pone.0012651)

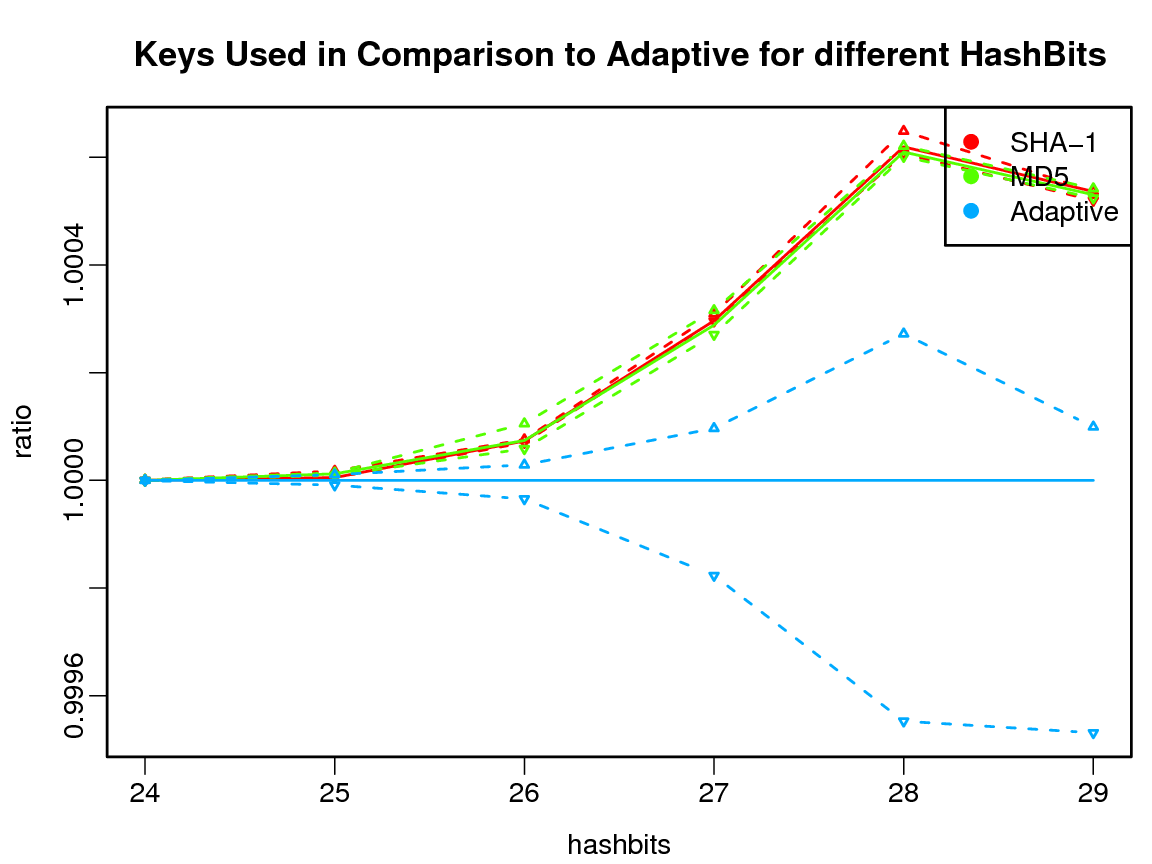

Supplement: Figure S1 — Hash keys used in comparison by SHA-1/MD5 hash algorithms in comparison to adaptive hashing at different hashbit values. This graph doesn't include First-N in order to examine, and adaptive hash results to examine the minute difference between Adaptive, SHA-1, and MD5. Only for large hash keys (high values of hashbits) does adaptive diverge significantly from SHA-1 and MD5, and even then the difference is minuscule. (0.05 MB TIF) [file pone.0012651.s001.tif]

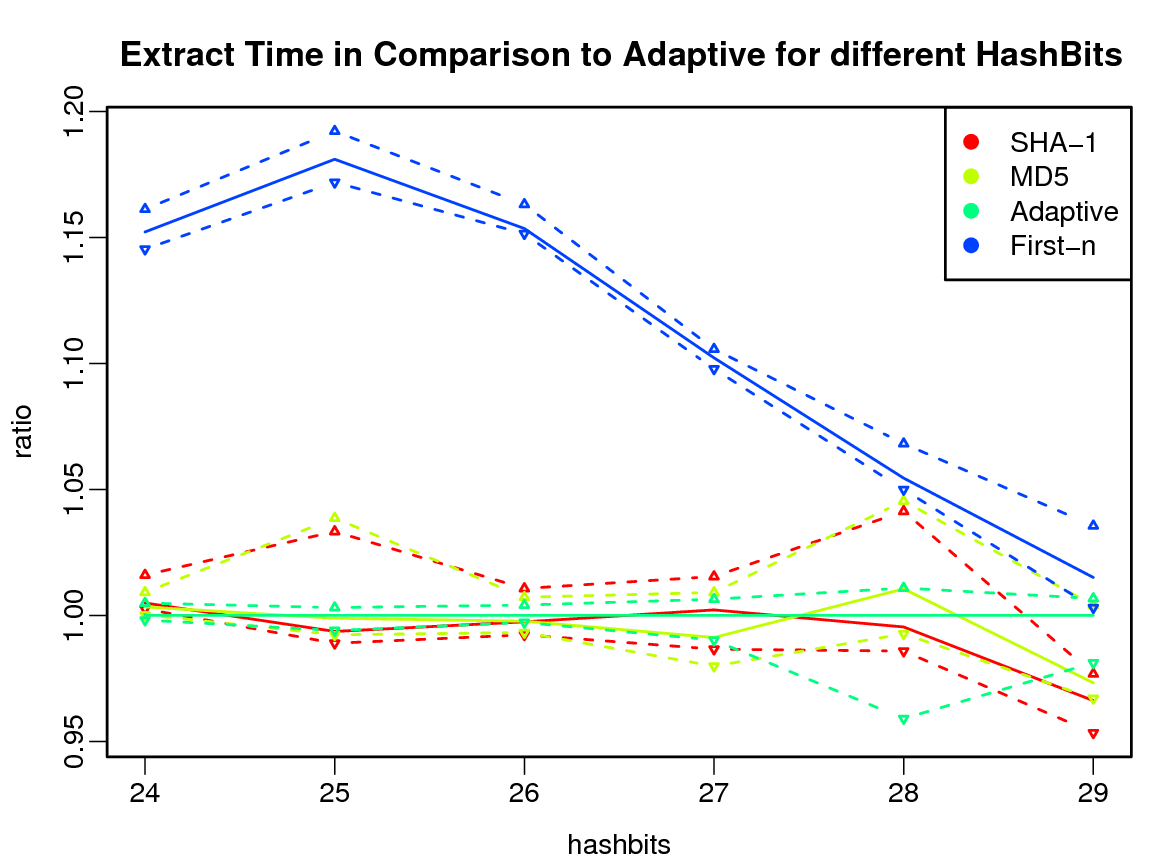

Supplement: Figure S2 — Extract time required by each hash algorithm compared to the adaptive hash algorithm. This graph shows the relative time required to extract matching seed sets from the hash table under different hash functions compared to the median time required our adaptive hash function. The solid line shows the median of all trials, while the dashed lines show the first and third quartiles. (0.06 MB TIF) [file pone.0012651.s002.tif]

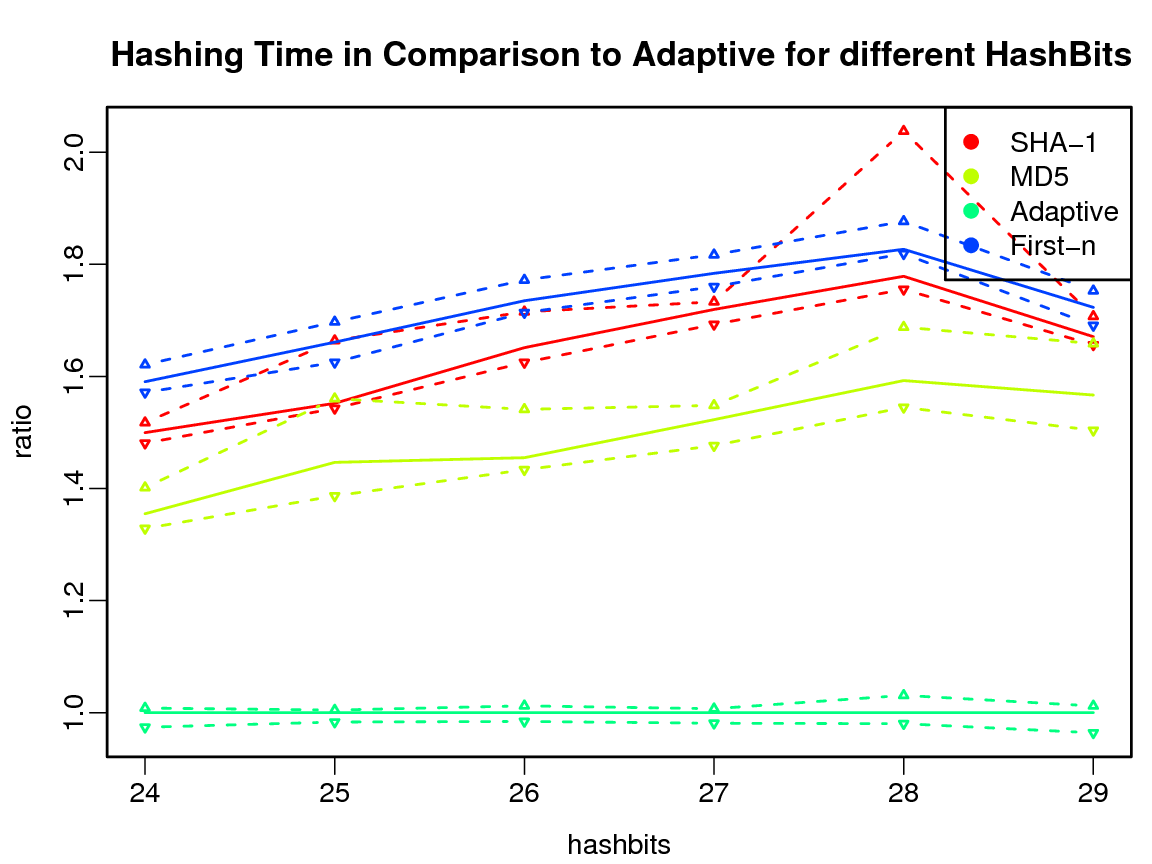

Supplement: Figure S3 — Time required to hash human and mouse X chromosomes using different hash functions at various hashbits settings compared to Adaptive. Here, we examine the difference in time required to compute hashes, store each (K,V) pair at different hashbits settings, again compared to our adaptive hash method. It's interesting to note that the naive First-N approach performs more poorly than even the slowest cryptographic hasher. (0.05 MB TIF) [file pone.0012651.s003.tif]

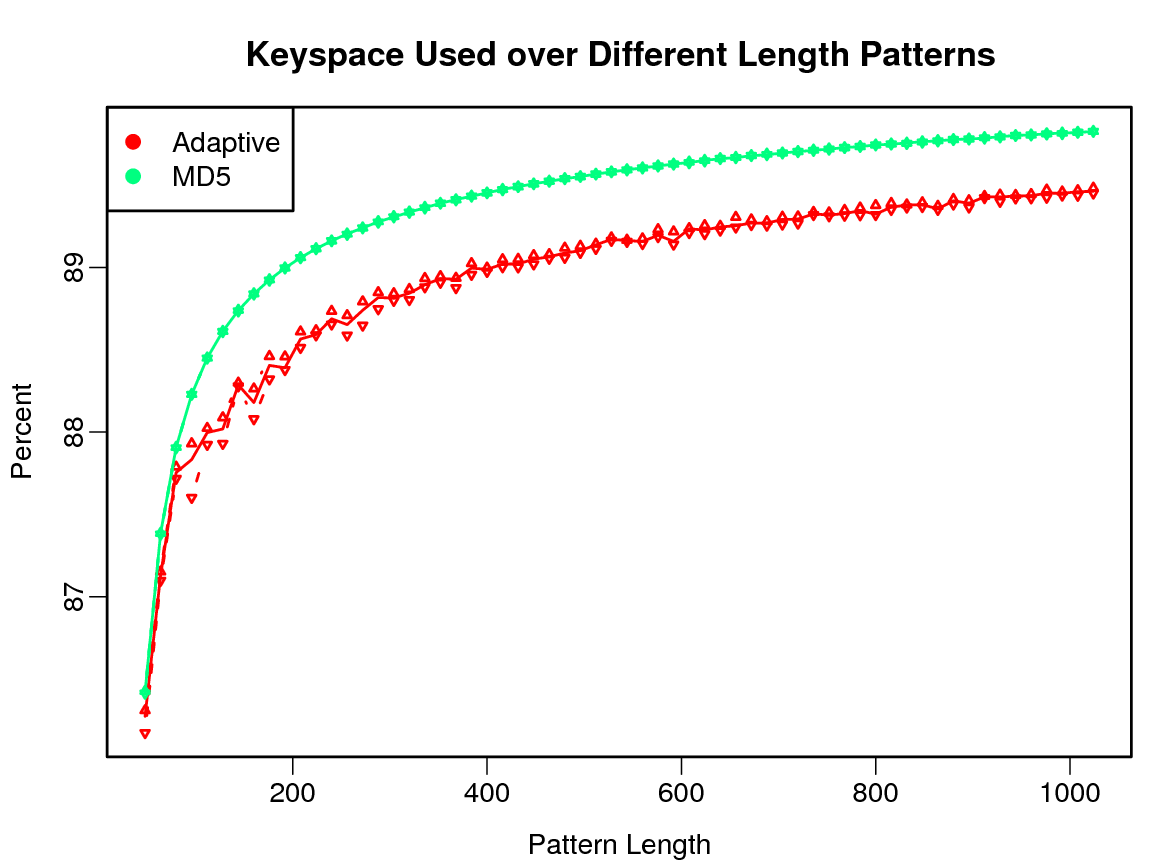

Supplement: Figure S4 — Comparing keyspace usage of Adaptive and MD5 hash functions for very long patterns. This graph shows the percent of possible hash keys produced by Adaptive and MD5 hash functions when hashing human and mouse X chromosomes. The number of hash keys possible increases with pattern length, because the number of observed unique seeds increases. Our adaptive hash algorithm keeps up with MD5 even for extremely long patterns. (0.05 MB TIF) [file pone.0012651.s004.tif]

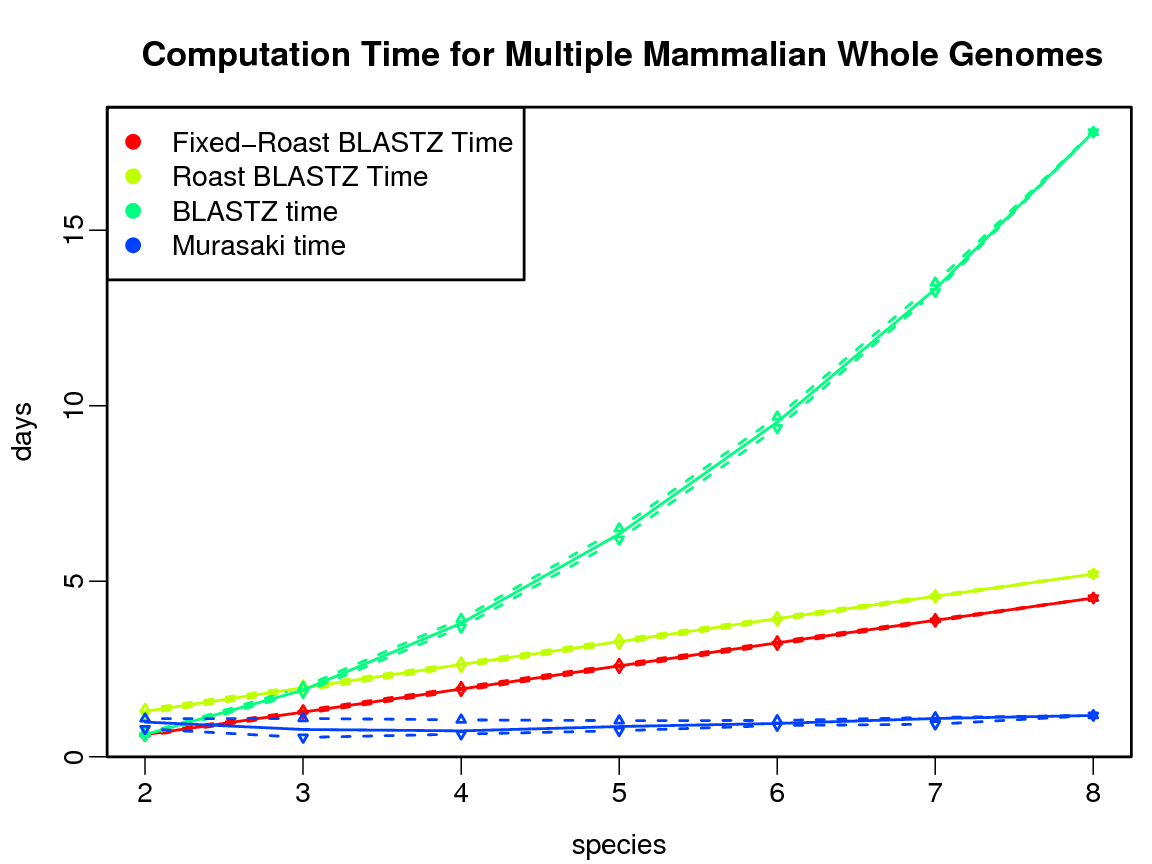

Supplement: Figure S5 — Computational time required to anchor multiple mammalian whole genomes. This graph shows the median CPU time in days required to anchor different numbers of mammalian whole genomes using TBA, Murasaki, and the patched and unpatched versions of Roast. The times for TBA and Roast include only the time spent on pairwise BLASTZ comparisons. The solid line represents the median of all tests for that number of species, while the dashed lines represent the first and third quartiles. (0.05 MB TIF) [file pone.0012651.s005.tif]

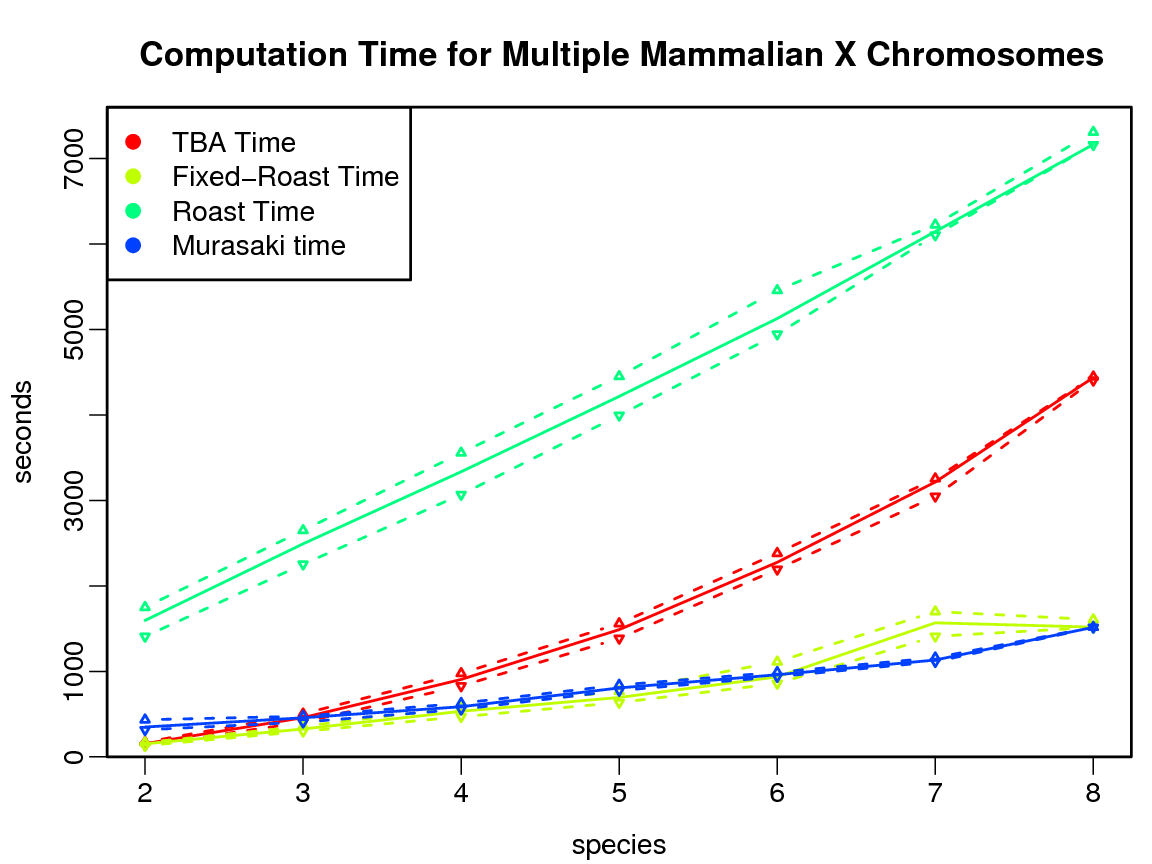

Supplement: Figure S6 — Computation time for multiple mammalian X chromosomes. This graph compares the computational time required to compare multiple X mammalian X chromosomes using Murasaki and the BLASTZ components of TBA, Roast, and our patched version of Roast. Because TBA requires all pairwise comparisons of the genomes under alignment, the time required for TBA grows quadratically, while Murasaki's time is nearly linear. The solid line represents the median of all tests for that number of species, while the dashed lines represent the first and third quartiles. (0.06 MB TIF) [file pone.0012651.s006.tif]
