# Supplementary material for: Murasaki: A Fast, Parallelizable Algorithm to Find Anchors from Multiple Genomes
Source: PLoS One. 2010 Sep 24;5(9):e12651. doi: 10.1371/journal.pone.0012651 (PMC2945767; doi:10.1371/journal.pone.0012651)
Supplement: Materials S1 — Additional detail on the implementation of hash functions, data structures, hash function fitness evaluation, pattern selection, runtime parameters, and our comparison with Roast. (0.39 MB PDF) [file pone.0012651.s007.pdf]

Supplemental Materials S1

1 Implementation

1.1 Hash Functions

Because the difference between the naive First-n approach and the others dwarfs the difference between any of the other differences, we look at only the 3 high-performing algorithms in Figure S1. We note that our adaptive hash algorithm starts to diverge only slightly when the key size increases above 25, and even then remains within 0.05% of the same number of keys as the cryptographic hashers, whereas the naive First-n approach is 32% behind any of the others.

We find that as more hash keys are used, fewer collisions require less work to invert the hash table, resulting in faster extraction times. Figure S2. We also measured the computational time required to hash the input sequences under each hash function, as shown in Figure S3.

The extraction times when using MD5 versus adaptive hash functions start to diverge slightly as pattern size increases. This divergence is a direct consequence of the divergence in hash key space utilization between the adaptive hasher and MD5 for very large patterns, as shown in Figure S4. The extent to which this divergence occurs and where it starts is in fact tunable via a parameter in the hash function generator genetic algorithm; however, for such long patterns we consider this divergence reasonable, as it has a minimal impact on the extraction time but a great savings on the hash time.

1.2 Implementation Details

Due to the wide variety of applications to which Murasaki is applied, we have built in a wide variety of user-tunable parameters to optimize Murasaki for various special purposes (such as hashing only every k bases as in BLAT [1] and PASH [2], defining alternative scoring/filtering parameters, or changing resource allocation when run in parallel). Most of the user-tunable parameters are determined automatically based on the input data and/or the user's computer environment. The main user-tunable parameter determining run time is the size of hash keys to use (which directly determines the hash table size), which we will call the "hashbits" parameter.

When storing hash keys, because K is the product of the location L and the "spaced seed pattern" parameter, K is determined implicitly by L and thus is never actually stored, but simply calculated as needed based on L.

1.3 Data structures

There are two important data structures within Murasaki.

1. The hash table structure

2. The anchor data structure

First, the hash table itself can be structured to use either chaining or open addressing. Under chaining, each entry of the hash table can contain values corresponding to multiple keys. When a collision occurs, where $K_i \neq K_j \wedge H(K_i) = H(K_j)$, then both corresponding L_i and L_j are stored at the same entry within the hash table, and disambiguated by some other means specific to the table entry structure. Under open addressing, the hash generated by $H(K_i)$ indicates the first place to *probe* for K_i. If some (K_j, L_j) pair is already stored there such that $K_i \neq K_j$ (i.e., a collision has occurred), then subsequent addresses are *probed* until either a matching K is found, or an empty address is found. There are many variations on how to probe, and Murasaki implements two (linear and quadratic probing [3]), but the idea remains the same. Open Addressing offers the advantage that there is no *sort* required to disambiguate non-matching seeds from within a single hash table entry. However a limitation of this design is that it is impossible to store more seeds than there are entries in the hash table. Murasaki automatically chooses whether to use chaining or open addressing based on the size of the input sequences (and thus the number of potential seeds) and the hash table size available.

The second key data structure is that for storing anchors themselves. Anchors are stored as a set of intervals indicating the beginning and end of the region anchored on each input sequence. These intervals are stored in Interval Trees [4]. Interval Trees provide a simple $O(\log A)$ method (where A is the number of anchors) to find overlapping regions, thereby facilitating the merging of anchors.

1.4 Hash function fitness

Determining the exact entropy content of a given hash function is a difficult problem with limited rewards compared with a good approximation; therefore, Murasaki employs an ad hoc method to estimate the entropy of the hash function. A bipartite graph is created consisting of entropy source nodes (the unmasked bases in the spaced seed pattern) and entropy sink nodes (each pair of bits in the hash key) where an edge is drawn if that base in the seed affects that bit in the hash. Each source node is allocated one unit of entropy (approximating the optimal 2 bits per base measure) that is then divided equally to weight the exiting edges. The entropy at each sink is then estimated as the summed weight of incoming edges, optionally modified by a "correlation penalty" in the range $(0, .2]$. The correlation penalty decreases inversely with the mean distance between source node pairs, with a maximum value of 0.2 assigned for adjacent bases. A correlation penalty like this is appropriate where input sequences are expected to conform to a Markov process (as is the case for DNA), but this may not be the case for all sequences. Finally, the estimated entropy is balanced against the computational cost of the function to determine the final fitness score.

2 Results

2.1 Pattern selection

We used a relatively arbitrary method for selecting the 24 base pattern (101111110101110111110011) used for tests comparing Murasaki to BLASTZ. We compared human and mouse X chromosomes using random patterns with lengths from 16 to 128. Patterns with lengths near 24 had sensitivity and specificity characteristics near that of BLASTZ while maintaining fast computational speed. We arbitrarily chose the highest scoring of our randomly generated length 24 patterns. We recognize that pattern choice is a complicated and important factor in the performance of ours and any other pattern-based homology search algorithm, and don't claim to have offered any better solutions to the problem of pattern choice, however our algorithm does provide a means run accurate searches using patterns selected by any method. We hope this provides a useful tool for experimenting with different pattern selection methods, and will look at ways to refine pattern selection in future work.

2.2 Murasaki Runtime Parameters

We also used the options "–mergefilter=100 and –scorefilter=6000" which is roughly equivalent to BLASTZ's "M=100 K=6000" options. "Mergefilter" limits the number of anchors that can be derived from any one seed to the number specified; any seeds which exceed this limit are tagged "repeats" and their locations are output separately. "Scorefilter" requires that all anchors have a minimum ungapped pairwise alignment score of at least the given threshold. For the exact meaning and usage of "mergefilter" and "scorefilter" see Murasaki's documentation. Furthermore, because the default TBA behavior is to pass its BLASTZ output through a program that removes all but the highest scoring regions among overlapping regions in a pair-wise comparison (similar to AxtBest in [5]), to provide a fair comparison, we filtered Murasaki's anchors the same way using the `align-best` tool provided in the Murasaki distribution.

The "mergefilter" filter alone can prevent the combinatorially explosive consequences of repeats, however in our mammalian sequence tests we use repeat masked sequences to reduce the amount of sequence hashed and stored in memory. We used the RepeatMasker [6] masked sequences in release 53 of the Ensembl genome database [7]. The genome sizes and fraction masked by RepeatMasker is shown in Table S1.

2.3 Pairwise Multiz with Roast

We also tested another alternative from the TBA package called Roast which appears to implement the method described in [8] which builds a multiple alignment based on pairwise comparisons between a reference sequence and all other sequences, thus in theory requiring time linear with respect to the number of sequences, similar to Murasaki. However due to an apparent bug in the implementation, Roast actually is actually worse than TBA in some cases. The bug causes comparisons of each sequence to itself to be included in the set

of pairwise comparisons performed for each alignment (for example "human-human" in a comparison of "human and mouse"). This is entirely unnecessary for the Multiz portion of the computation, but generally takes more than any other pair because of the large number of matches. This bug is fairly simple to fix, however may be beyond the reach of biologists without some programming ability, therefore we tested both versions. The results are shown in Figure S5. Because BLASTZ cannot handle whole genomes in a single pass, input has to be broken up into chromosome sized fragments. This means that what was a comparison of "human x mouse" in Murasaki, becomes a comparison of "human-1 x mouse-1 + human-1 x mouse-2 + ... human-1 x mouse-M + human-2 x mouse-1 + human2 x mouse-2 + ... human-M x mouse-M." Each comparison is shorter (N/M in size), however we now do M^2 times as many, requiring $O(NM)$ time. However, because M is forced by the constraints of the system to be N/S where S is the maximum size the computer system can handle, $O(M) = O(N/S) = O(N)$ and therefore the asymtotic time requirements of Roast must be $O(N^2)$. The behavior of Roast on small sequences, on the other hand, is nearly identical to Murasaki, and is shown in Figure S6.

References

1. Kent WJ (2002) BLAT—The BLAST-Like Alignment Tool. Genome Res 12: 656-664.

2. Ken J Kalafus AM Andrew R Jackson (2004) Pash: Efficient genome-scale sequence anchoring by positional hashing. Genome Research 14: 672-678.

3. Knuth DE (1973) Sorting and Searching, volume 3 of The art of computer programming. Addison Wesley.

4. Cormen T, Leiserson C, Rivest R (1990) Introduction to Algorithms. MIT Press.

5. Schwartz S, Kent WJ, Smit A, Zhang Z, Baertsch R, et al. (2003) Human-Mouse Alignments with BLASTZ. Genome Res 13: 103-107.

6. Smit A, R H, Green P (1996-2004). Repeatmasker open-3.0. URL http://www.repeatmasker.org.

7. Hubbard T, Barker D, Birney E, Cameron G, Chen Y, et al. (2002) The Ensembl genome database project. Nucl Acids Res 30: 38-41.

8. Miller W, Rosenbloom K, Hardison RC, Hou M, Taylor J, et al. (2007) 28-way vertebrate alignment and conservation track in the UCSC Genome Browser. Genome Res 17: 1797–1808.

3 Figures

4 Tables

Table S1 - Genome Sizes

This table shows the sequence sizes used in the mammalian whole genome and comparisons and the respective fractions masked by RepeatMasker [6].

Species	Total Bases (MB)	Masked Bases (MB)	Fraction masked
Human	2855.344	1434.406	0.502
Chimp	2752.356	1402.532	0.510
Rhesus	2646.263	1375.097	0.520
Orangutan	2722.968	1348.973	0.495
Mouse	2558.509	1443.026	0.564
Rat	2477.054	1386.647	0.560
Dog	2309.875	1367.003	0.592
Cow	2466.956	1319.883	0.535

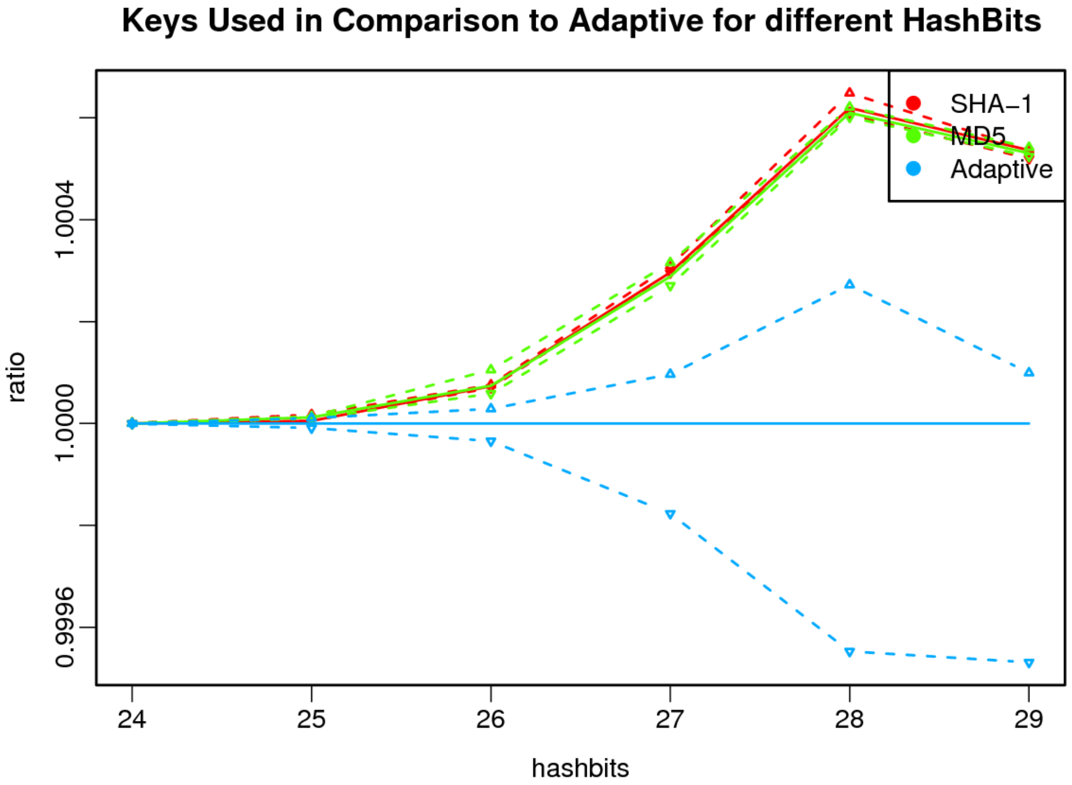

Figure S1. Hash keys used in comparison by SHA-1/MD5 hash algorithms in comparison to adaptive hashing at different hashbit values.
This graph doesn't include First-N in order to examine, and adaptive hash results to examine the minute difference between Adaptive, SHA-1, and MD5. Only for large hash keys (high values of hashbits) does adaptive diverge significantly from SHA-1 and MD5, and even then the difference is minuscule.

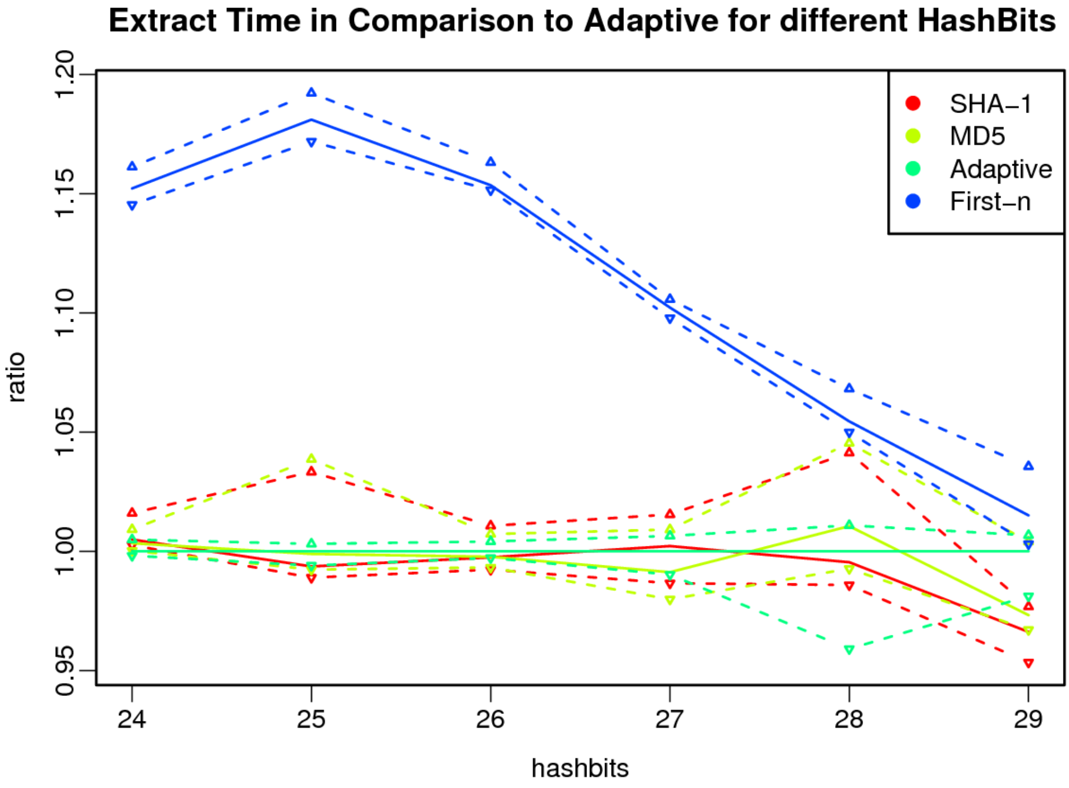

Figure S2. Extract time required by each hash algorithm compared to the adaptive hash algorithm.
This graph shows the relative time required to extract matching seed sets from the hash table under different hash functions compared to the median time required our adaptive hash function. The solid line shows the median of all trials while the dashed lines show the first and third quartiles.

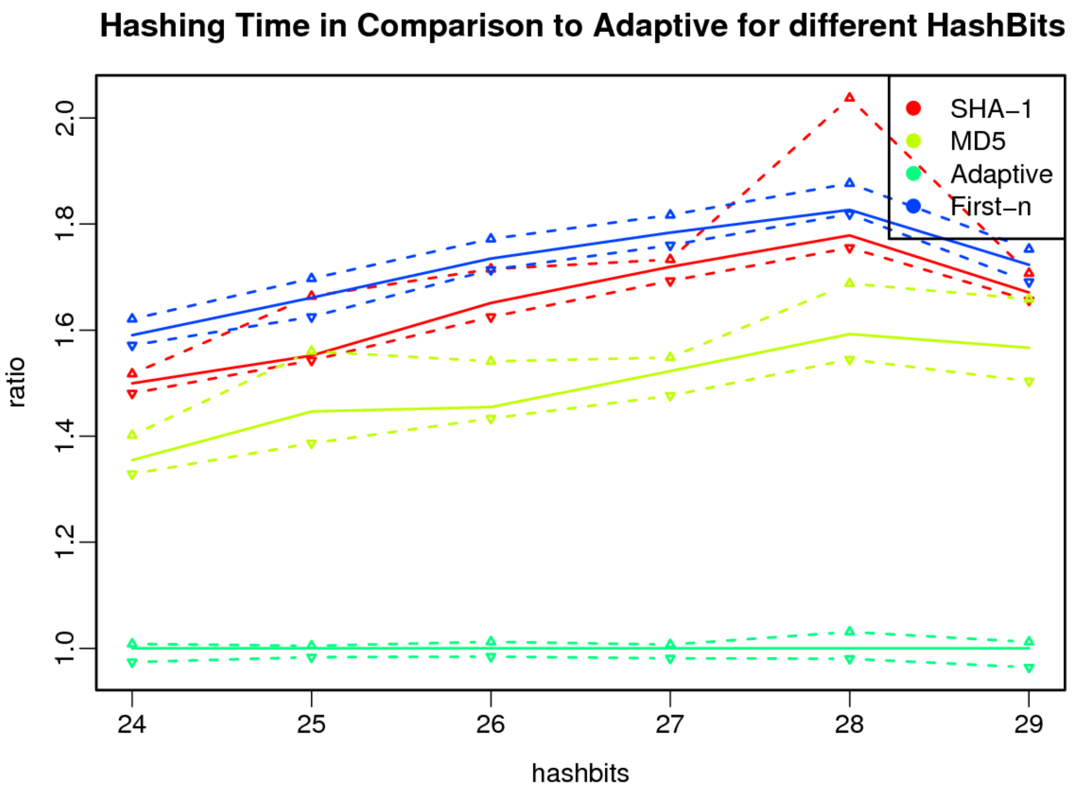

Figure S3. Time required to hash human and mouse X chromosomes using different hash functions at various hashbits settings compared to Adaptive.
Here we examine the difference in time required to compute hashes store each (K, V) pair at different hashbits settings, again compared to our adaptive hash method. It's interesting to note that the naive First-n approach performs more poorly than even the slowest cryptographic hasher.

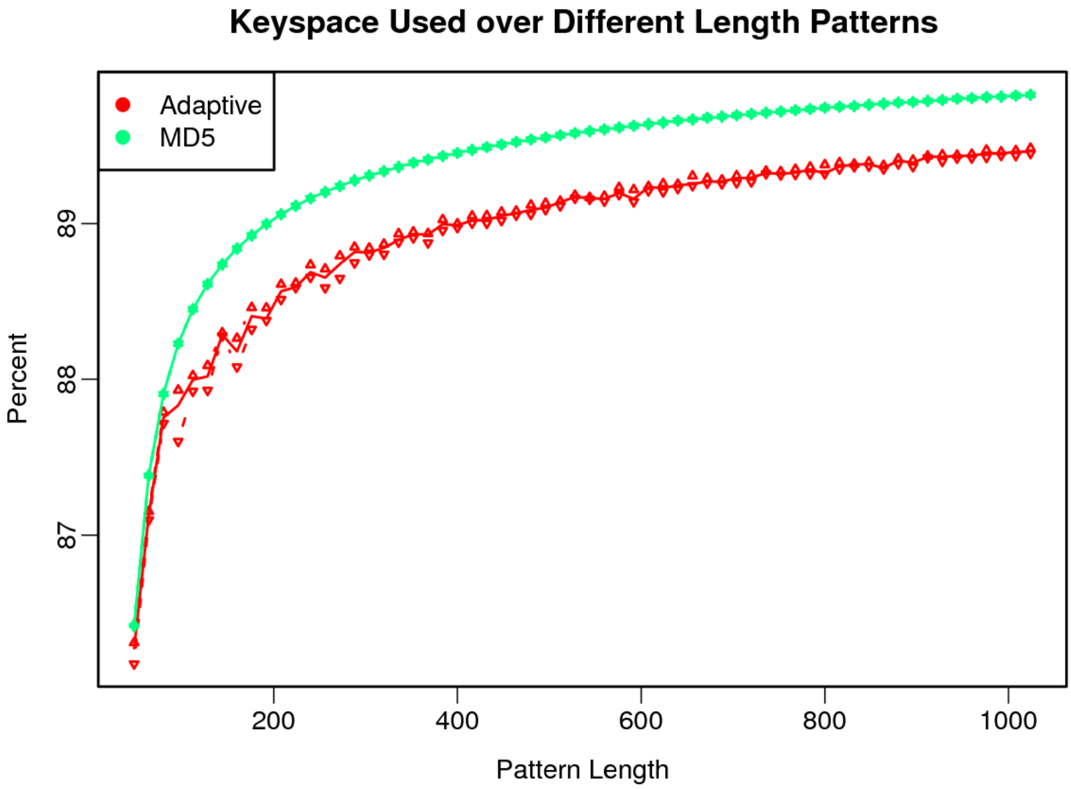

Figure S4. Comparing Keyspace Usage of Adaptive and MD5 Hash Functions For Very Long Patterns.
This graph shows the percent of possible hash keys produced by Adaptive and MD5 hash functions when hashing Human and Mouse X chromosomes. The number of hash keys possible increases with pattern length because the number of observed unique seeds increases. Our adaptive hash algorithm keeps up with MD5 even for extremely long patterns.

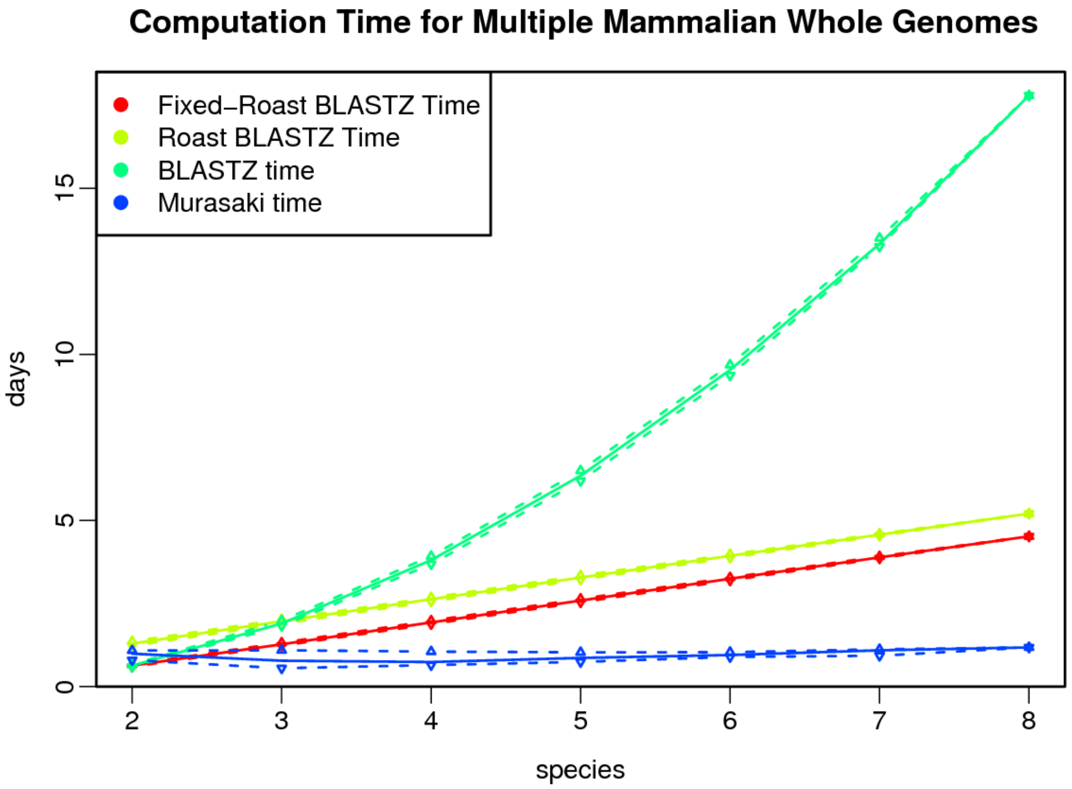

Figure S5. Computational time required to anchor multiple mammalian whole genomes. This graph shows the median CPU time in days required to anchor different numbers of mammalian whole genomes using TBA, Murasaki, and the patched and unpatched versions of Roast. The times for TBA and Roast include only the time spent on pairwise BLASTZ comparisons. The solid line represents the median of all tests for that number of species, while the dashed lines represent the first and third quartiles.

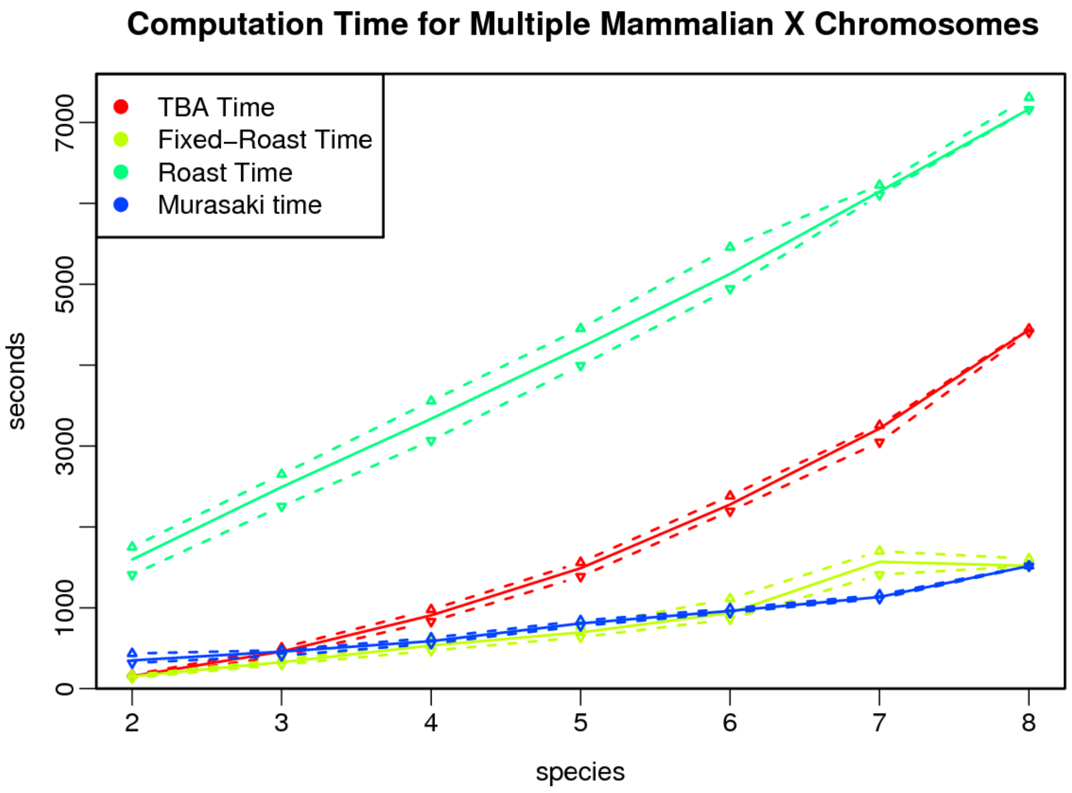

Figure S6. Computation Time for Multiple Mammalian X Chromosomes. This graph compares the computational time required to compare multiple X mammalian X chromosomes using Murasaki and the BLASTZ components of TBA, Roast, and our patched version of Roast. Because TBA requires all pairwise comparisons of the genomes under alignment, the time required for TBA grows quadratically, while Murasaki's time is near linear. The solid line represents the median of all tests for that number of species, while the dashed lines represent the first and third quartiles.
